# One of the major challenges of masking the bitter taste in medications: an overview of quantitative methods for bitterness

**DOI:** 10.3389/fchem.2024.1449536

**Published:** 2024-08-14

**Authors:** Panpan Wang, Haiyang Li, Yanli Wang, Fengyu Dong, Han Li, Xinjing Gui, Yanna Ren, Xiaojie Gao, Xuelin Li, Ruixin Liu

**Affiliations:** ^1^ Department of Pharmacy, The First Affiliated Hospital of Henan University of Chinese Medicine, Zhengzhou, China; ^2^ School of Pharmacy, Henan University of Chinese Medicine, Zhengzhou, China; ^3^ Henan Province Engineering Research Center for Clinical Application, Evaluation and Transformation of Traditional Chinese Medicine, Henan Province Engineering Laboratory for Clinical Evaluation Technology of Chinese Medicine, Zhengzhou, China; ^4^ Collaborative Innovation Center for Chinese Medicine and Respiratory Diseases Co-Constructed by Henan Province, Education Ministry of China, Henan University of Chinese Medicine, Zhengzhou, China; ^5^ Zhengzhou Traditional Chinese Medicine Hospital, Zhengzhou, China; ^6^ Third Level Laboratory of Traditional Chinese Medicine Preparations of the State Administration of Traditional Chinese Medicine, Zhengzhou, China

**Keywords:** bitterness, quantitative method, traditional human taste panel method, active pharmaceuticals ingredients, traditional Chinese medicine

## Abstract

**Objective:**

The aim of the present study was to carry out a systematic research on bitterness quantification to provide a reference for scholars and pharmaceutical developers to carry out drug taste masking research. Significance: The bitterness of medications poses a significant concern for clinicians and patients. Scientifically measuring the intensity of drug bitterness is pivotal for enhancing drug palatability and broadening their clinical utility.

**Methods:**

The current study was carried out by conducting a systematic literature review that identified relevant papers from indexed databases. Numerous studies and research are cited and quoted in this article to summarize the features, strengths, and applicability of quantitative bitterness assessment methods.

**Results:**

In our research, we systematically outlined the classification and key advancements in quantitative research methods for assessing drug bitterness, including *in vivo* quantification techniques such as traditional human taste panel methods, as well as *in vitro* quantification methods such as electronic tongue analysis. It focused on the quantitative methods and difficulties of bitterness of natural drugs with complex system characteristics and their difficulties in quantification, and proposes possible future research directions.

**Conclusion:**

The quantitative methods of bitterness were summarized, which laid an important foundation for the construction of a comprehensive bitterness quantification standard system and the formulation of accurate, efficient and rich taste masking strategies.

## 1 Introduction

As widely recognized, the axiom “good medicine tastes bitter” epitomizes a fundamental attribute of pharmaceuticals, with many drugs exhibiting a bitter taste (Bahia et al., 2018). Our investigation revealed that bitter herbs or decoction pieces constituted 49.0% of the 2020 edition of the “Chinese Pharmacopoeia" (Lin et al., 2016), while 66% of compounds cataloged in the Drug Bank library were projected to possess a bitter taste (Dagan-Wiener et al., 2017). The Bitter DB database archives over 1,000 bitter molecules. Humans and animals exhibit heightened sensitivity to bitterness perception, capable of discerning bitterness even at lower concentrations (Aliani and Eskin, 2017). The intrinsic aversion to bitterness among humans significantly impacts patient medication adherence (Beauchamp, 2016; Boesveldt and de Graaf, 2017), thereby influencing clinical efficacy (Amin et al., 2018; Zheng et al., 2018). The prevalent distaste for medications is frequently cited as a primary reason for patient non-compliance, particularly among children (Clapham et al., 2023). Surveys indicate that over 90% of pediatricians identify drug taste and palatability as major barriers to completing clinical treatments (Milne and Bruss, 2008). In a survey involving nearly 700 European children, 63.7% of respondents attributed difficulty in medication intake to dislike for the drug’s taste (Nordenmalm et al., 2019). Peter Drucker, often regarded as the father of modern management, emphasized the necessity of objective and accurate quantitative evaluation of drug bitterness as a crucial prerequisite for understanding its taste patterns and enhancing palatability.

**FIGURE 1:**
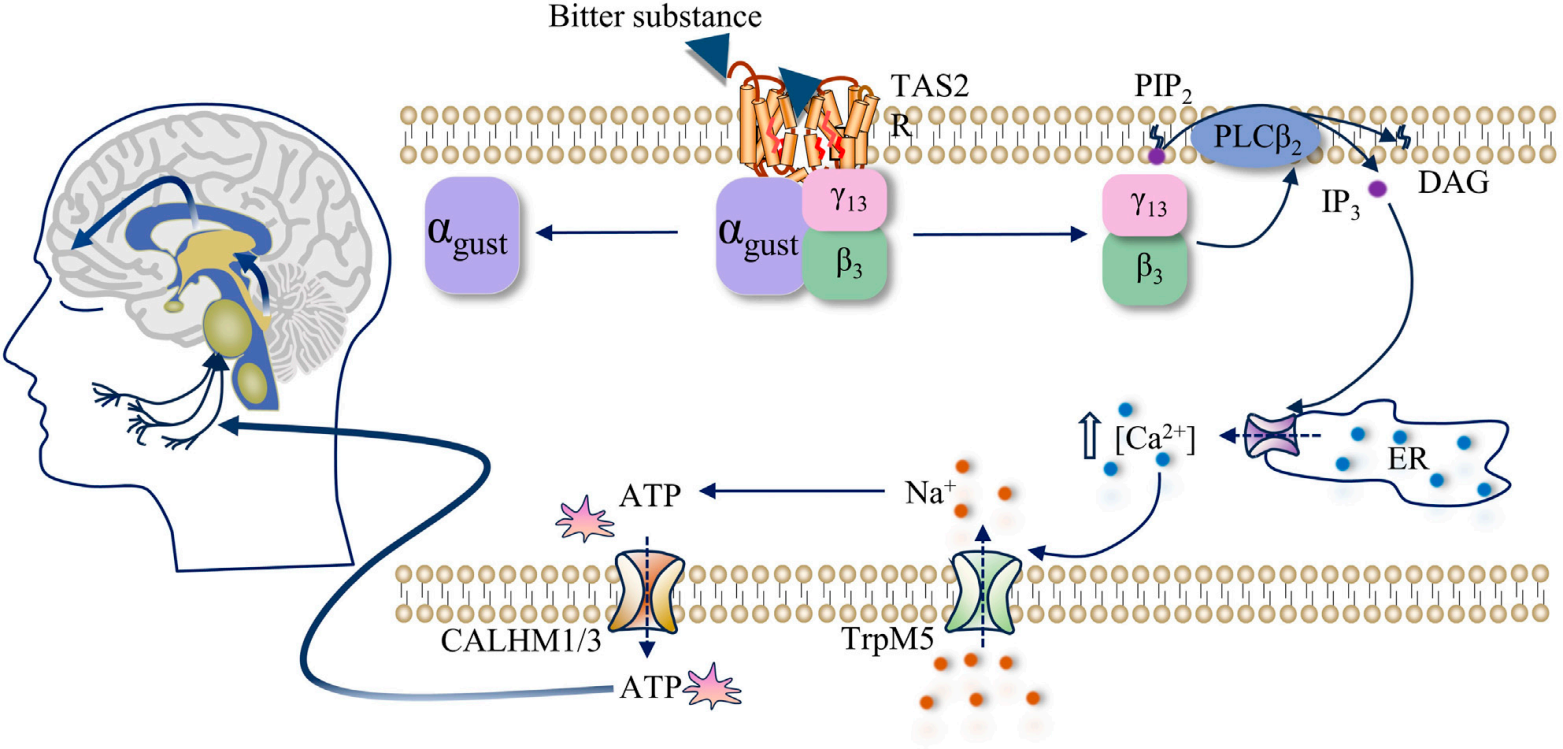
Activation mechanism of human TAS2R and signal transduction pathway of bitterness stimulation.

Bitterness primarily arises from the activation of TAS2R (also referred to as bitter receptors). Upon binding of the bitter compound to the receptor, located prominently on taste receptor cells (TRCs), a signal transduction cascade ensues. This activation prompts TAS2R to catalyze the dissociation and liberation of Gβ3/Gγ13 subunits from the Gβ3/Gγ13 heterotrimeric receptor, thereby activating phospholipase C (PLCβ2). Subsequently, PLCβ2 catalyzes the breakdown of phospholipid PIP2, yielding inositol-1,4,5-phosphate (IP3). IP3, in turn, binds to IP3 receptors on the endoplasmic reticulum, eliciting the release of intracellularly stored calcium ions (Ca^2+^). The elevated intracellular Ca^2+^ concentration ([Ca^2+^]i) prompts the opening of membrane-associated TRPM5 channels, facilitating the influx of sodium ions (Na^+^). This ion exchange initiates receptor cell membrane depolarization, triggering the release of adenosine triphosphate (ATP) through calcium homeostasis regulator one and 3 (CALHM1 and CALHM3) channels. Ultimately, the liberated ATP activates purinergic receptors on afferent nerve fibers, converting the chemical signals of bitter compounds into electrical signals, which are relayed to the taste nucleus of the brainstem. Subsequently, these signals are transmitted to the thalamus and eventually to the taste cortex of the cerebral cortex, culminating in the perception of bitterness (Finger et al., 2005; Roper, 2007; Ma et al., 2018), as shown in Figure 1.

According to the law of taste and bitter perception, the factors affecting drug bitterness are affected by the difference of genes and receptors. Single nucleotide polymorphisms (SNPs) in genes encoding bitter taste receptors (TAS2R) leading to varied responses to bitter stimuli. Consequently, individuals with one gene form may perceive strong bitterness, while those with another gene form may not perceive bitterness at all (Hayes et al., 2011; Mennella et al., 2011; Roudnitzky et al., 2011; Roudnitzky et al., 2015; Nolden and Feeney, 2020). The heterologous functional expression experiments have revealed that 25 TAS2Rs exhibit varying degrees of regulation characteristics as bitter taste receptors (Meyerhof et al., 2010). The ability of bitter compounds to stimulate TAS2Rs varies. Compared to the activation of a single receptor, simultaneous activation of multiple receptors by a compound can elicit a larger cellular or neural response, thereby increasing bitterness. Additionally, the factors that affect the bitterness also include the following: (1) Chemical structure. According to the three-point contact theory of bitterness formation, bitter molecules can be categorized into two groups (Hans-Dieter et al., 1985; Meyerhof, 2005): one consisting of hydrophobic lipophilic compounds with limited water solubility, such as olefins and terpenoids; the other comprising highly polar compounds capable of forming robust hydrophobic interactions, exemplified by alkaloids (Zeng, 1990). (2) Substance concentration. Within a specific concentration range of the same drug, bitterness demonstrates a positive correlation with concentration. Studies have shown that a logarithmic relationship between human sensory intensity and stimulus physical quantity under moderate intensity stimula56tion conditions (S = KlgR) (Omür-Ozbek and Dietrich, 2008; Li et al., 2016). (3) Interaction between substances. The bitterness between substances can be affected by electrostatic interaction (Schalk et al., 2018), hydrophobic interaction (Ogi et al., 2015), covalent bonding (Bohin et al., 2013), and inclusion interaction (Shah and Mashru, 2008). (4) pH. It is established that certain acidic peptides can mitigate bitterness (Sakurai et al., 2009). Bitterness inhibition of sesquiterpene lactone can be achieved by pH control (Yanagisawa and Misaka, 2021). (5) Solution viscosity. Studies have found that an increase in the viscosity of the resulting aqueous solvent leads to a reduction in taste intensity. Additionally, studies have indicated that emulsions demonstrate bitterness inhibition effects on KCl and/or caffeine compared to aqueous solutions (Torrico et al., 2015).

In the realm of food taste assessment, extensive studies have focused on quantitatively evaluating taste attributes. For instance, the bitterness of beer is commonly assessed based on the concentration of isomerized α-acids, the primary source of beer bitterness. Methods such as European Bitterness Units (EBU) (Polshin et al., 2010; Rudnitskaya et al., 2010), International Bitterness Units (IBU) (Howard, 1968; Donley and Anheuser, 1992; Christensen et al., 2005), E.B.C. Bitterness Units (Bishop, 2013), and Bitterness Units (BU) (Tomlinson et al., 2013) are utilized for this purpose. Caffeine, containing numerous bitter compounds, undergoes bitterness intensity evaluation using Sensory Lexicon (Shibamoto et al., 1981; Ginz and Engelhardt, 2000; Aree, 2019). These established quantification methods in the field of food taste can serve as valuable references for assessing drug bitterness. While the bitterness of chemical drugs can be accurately measured based on the content of bitter compounds due to their clear and singular composition, natural drugs encompass a multitude of bitter substances, intricate substance interactions, and a diverse array of taste components. These complexities confer inherent bitterness to natural drugs, emphasizing the importance of elucidating the mechanisms underlying their bitter taste and exploring tailored quantification methodologies.

As research into drug palatability continues to evolve, investigators have undertaken studies on the measurement and quantitative assessment of drug bitterness (Gaudette and Pickering, 2013). These studies primarily encompass *in vivo* and *in vitro* methods. *In vivo* evaluation methods include the traditional human taste panel method (THTPM) (Miyanaga et al., 2003; Rudnitskaya et al., 2013), taste strips test method (Liu et al., 2020), animal behavior tests (Lemon et al., 2019), and facial expression analysis (Lemon et al., 2019), with THTPM recognized as the gold standard for taste assessment (Gunaratne et al., 2019). *In vitro* bitterness detection methods mainly consist of electronic tongue methods (ETM) (Hui et al., 2014; Immohr et al., 2017), and cell-based evaluations (Qingjun and Ping, 2009), among others. Following an understanding of drug bitterness, researchers have employed taste-masking techniques utilizing flavoring agents, bitterness inhibitors, cyclodextrins, and nanoemulsions (Hu et al., 2023). These methods have contributed positively to advancing the objective measurement and precise control of bitterness, thereby enhancing clinical drug compliance. Nonetheless, despite these advancements, there remains a lack of systematic summarization of research methods for bitterness quantification, as each method possesses unique characteristics and applications. This article seeks to analyze the research progress in drug bitterness quantification, delineate the primary factors influencing drug bitterness, and compile the methodologies for bitterness quantification. The aim is to foster a systematic comprehension of the principles, methodologies, and attributes of bitterness quantification, thereby offering insights for research endeavors in areas such as drug bitterness intensity evaluation, taste masking, and related fields.

## 2 Quantitative methods for bitterness

In light of the various factors influencing drug bitterness as outlined above, researchers are continuously innovating quantitative methods for assessing drug bitterness. Broadly, these methods can be categorized into two types: one involves quantifying the bitterness of drugs by measuring bitter molecules, while the other quantifies drug bitterness based on the intensity of taste stimulation. The results of both methods are elaborated upon below.

### 2.1 THTPM

**FIGURE 2:**
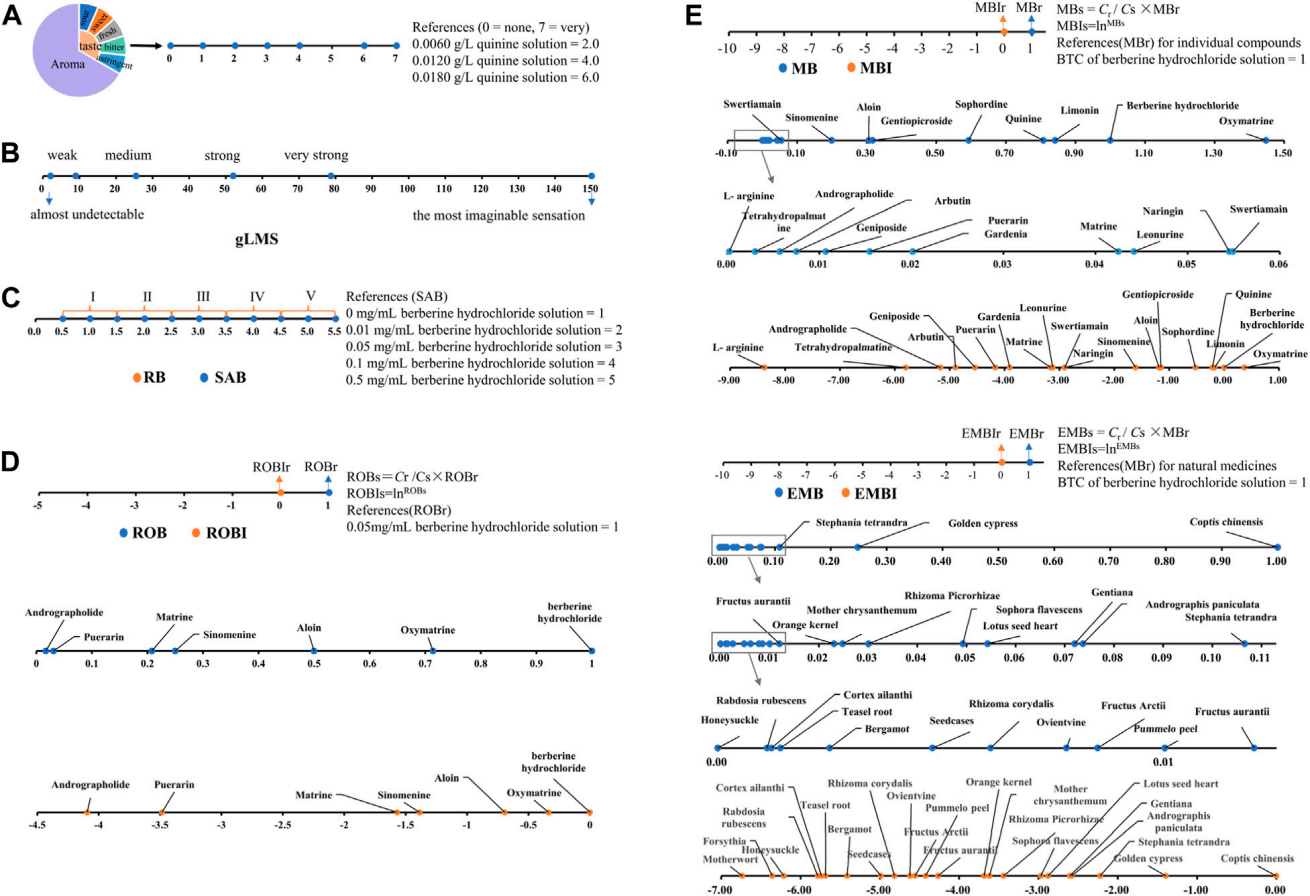
Quantitative research method of bitterness based on the THTPM. **(A)** Schematic representation of the quantitative description analysis (QDA). Based on Huan-Huan Li, 2019 (Li et al., 2019). **(B)** Schematic representation of the general labeled magnitude Scale (gLMS) (Deng et al., 2022) **(C)** Schematic representation of Rank bitterness intensity (RB) and standard apparent bitterness (SAB). Representative images were our own work (Li et al., 2023). **(D)** Schematic representation of Ratio of bitterness (ROB). Representative images were our own work (Gao et al., 2022). **(E)** chematic representation of Molecular bitterness (MB) and equivalent molecular bitterness (EMB). Representative images were our own work (Wang et al., 2022).

THTPM is a method used to assess the taste of drugs or food, relying on specific technical specifications and processes and utilizing the taste sense of the evaluation group (Medicine, 2024). This method falls under the category of quantifying bitterness based on the intensity of human taste stimulus. The main methods were illustrated in Figure 2. Currently, a range of internationally recognized standards for quantitative sensory evaluation have been established (Clapham et al., 2023), paving the way for researchers to conduct various explorations into quantitative bitterness assessment.

#### 2.1.1 Quantitative description analysis (QDA)

QDA is a method used to characterize the sensory properties and intensity of drugs. Within QDA, standard reference materials provide the bitterness value against which samples are compared for evaluation. For instance, when assessing the bitterness of Huanglian Jiedu Decoction (HLJDD), scholars provide a reference standard of 0.2 g/mL HLJDD with a bitterness value of 10. Evaluators use this reference standard to assess the bitterness of HLJDD after masking with [mPEG2000-PLLA2000, γ-CD, and neotame], revealing a reduction in HLJDD bitterness (Ke et al., 2022).

However, the methods mentioned above lack the ability to provide information on the temporal aspects of bitterness perception. Bitterness exhibits unique temporal characteristics, taking more time to reach peak intensity in the mouth and longer to return to baseline (Guinard et al., 2010). Additionally, with repeated intake, the perceived intensity of bitterness tends to increase (Guinard et al., 1986). The presence of polyphenols (in red wine) and isohumulone (in beer) may amplify the bitterness of these beverages during consumption (Guinard et al., 1994; Noble, 1994). Hence, some scholars employ the time-intensity method to dynamically assess bitterness throughout the entire perception period. In a study evaluating the bitterness of berberine hydrochloride orally disintegrating tablets, researchers instructed subjects to record bitterness intensity levels at various intervals (30 s, 1 min, 2 min, 5 min, 10 min) within a span of 10 min. Bitterness intensity was rated on a scale from 0 to 3. The findings indicated that when the ratio of the drug to the pH-dependent polymer Eudragit E100 increased from 1:0 to 1:0.8, the bitterness of the drug microcapsules significantly decreased, reaching zero bitterness by the second minute (Hu et al., 2013).

Quantitative descriptive analysis also encompasses the evaluation of bitterness across different taste categories. Some researchers (Sook Chung and Lee, 2012) categorized bitterness into distinct types such as alcohol bitterness, coffee bitterness, grapefruit pith bitterness, medicinal bitterness, and cocoa bitterness. Each type of bitterness corresponds to unique definitions and references, posing higher demands on the evaluator’s assessment skills.

#### 2.1.2 General labeled magnitude scale (gLMS)

The gLMS is a psychophysical scale used for sensory testing to evaluate the taste and aftertaste of various stimuli. It comprises a 150 mm line spanning from the bottom to the top of the vertical axis. The scale includes descriptors such as “almost undetectable” (2.1 mm; 1.4 units), “weak” (9 mm; 6 units), “medium” (25.5 mm; 17 units), “strong” (52.05 mm; 34.7 units), “very strong” (78.75 mm; 52.5 units), and “the most imaginable sensation” (150 mm; 100 units). The scale presents adjectives to evaluators without numerical values. Experimenters place the adjectives on the scale in a semi-logarithmic manner based on determined intervals to generate data equivalent to magnitude estimation (Green et al., 1993; Green et al., 1996). Subsequently, numerical data are calculated based on the scale. Using the gLMS method, the intensity of different bitter substances can be directly compared. For instance, Deng (Deng et al., 2022) employed the gLMS to conduct sensory tests on adults, comparing the bitterness intensity of prednisolone solution with that of quinine. The results indicated that the bitterness level of prednisolone saturated solution (average gLMS score: 46.8) was similar to that of a 1 mM quinine solution (average gLMS score: 40.1). However, substantial individual differences may exist in gLMS assessment. For example, researchers measured the minimum and maximum values of 1 mM quinine as 8.7 and 90.0, respectively (Deng et al., 2022). Furthermore, variations in sensory test data among different researchers also occur. While one study (Deng et al., 2022) reported the average gLMS score of 1 mM quinine as 40.1, another study (Cruickshanks et al., 2009) documented a gLMS score of 50 at the same concentration. Hence, stringent and standardized conditions are necessary for bitterness evaluation using this method.

#### 2.1.3 Rank bitterness intensity (RB) and standard apparent bitterness (SAB)

In bitterness evaluation, descriptions like “unbearable bitterness,” “a little bitterness,” and “almost no bitterness” often arise, necessitating a method for grading bitterness. In a study on chlorphenamine maleate bitterness, researchers categorized bitterness into five levels: (A) 5: very strong bitterness, (B) 4: strong bitterness, (C) 3: medium bitterness, (D) 2: slightly bitter, and (E) 1: tasteless. Using the uncomplexed pure drug as a control with an average bitterness value of 5, subjects were instructed to compare the bitterness of different drug resin complexes (DRC) with the control and express the perceived bitterness level. The findings revealed that Indion-234, Tulsion-343, and Tulsion-344 effectively masked the bitterness of chlorphenamine maleate, with the bitterness of the drug in DRC decreasing as the ratio of drug to resin increased (Yewale et al., 2013).

Inspired by this approach, some scholars introduced the concept of “RB” (Wang et al., 2021), using berberine (BBR) as a reference. After volunteers pre-tested multiple concentrations, different concentrations of BBR corresponding to each bitterness level were determined (Table 1). The practical application of graded bitterness involves evaluating unknown bitterness samples by referencing the bitterness level and value range of the reference sample group. Once graded, the specific bitterness value is assigned according to the corresponding bitterness range of each grade. Bitterness determined for the reference material in the solution state is termed “Standard Reference Bitterness (SRB)" (Liu et al., 2019; Zhang et al., 2021). Bitterness of other drugs established based on SRB as a reference in the solution state is referred to as “Standard Apparent Bitterness (SAB)", and the level of standard apparent bitterness is known as “Standard Apparent Rank Bitterness (SARB)". Employing these methods enables us to comprehend the bitterness levels and bitterness profiles of different bitter substances.

**TABLE 1:** Bitterness ranking and concentration of corresponding reference samples.

No.	Description of bitterness intensity	RB	Corresponding scale	Conc. Of reference samples	SRB
1	Imperceptible	Ⅰ	[0.5, 1.5)	0 mg/mL (0 mM)	1
2	Slight	Ⅱ	[1.5, 2.5)	0.01 mg/mL (0.027 mM)	2
3	Moderate	Ⅲ	[2.5, 3.5)	0.05 mg/mL (0.134 mM)	3
4	High (but still acceptable)	Ⅳ	[3.5, 4.5)	0.1 mg/mL (0.269 mM)	4
5	Extreme (almost unacceptable)	Ⅴ	[4.5, 5.5]	0.5 mg/mL (1.344 mM)	5

Shi et al. (2013) utilized a berberine hydrochloride aqueous solution as the reference solution and applied the THTPM method to assess the bitterness grade of six notable bitter Chinese herbal decoction pieces, including Cortex Phellodendri, Radix Gentianae, Herba Andrographis, Radix Ginseng, and Nelumbinis Plumulae. They investigated the taste-masking effect of β-CD at various mass fractions. Results indicated that the taste-masking effect improved with increasing β-CD concentration. With the exception of Cortex Phellodendri, the bitterness of the liquid after adding 3% β-CD was within a low range (0.65 ± 0.05), all falling into the almost no bitterness grade. This suggested that β-CD could effectively mask the bitter taste of traditional Chinese medicine. (Li et al., 2011). employed the THTPM method to assess the water decoction of 35 different single Chinese medicine decoction pieces with varying bitterness, using berberine hydrochloride as a reference. They preliminarily obtained the bitterness value and distribution characteristics of the water decoction of Chinese medicine decoction pieces, providing a crucial foundation for subsequent taste-masking research (Liu et al., 2012; Liu et al., 2013). conducted research on matrine at different concentrations, with 20 evaluators assessing its bitterness level and specific standard apparent bitterness value. They evaluated sample bitterness using three methods: the order evaluation method (OEM ranking method), score evaluation method (SEM), and integrated score evaluation method (ISEM). Ultimately, the three methods were comprehensively analyzed based on sorting accuracy, judgment sensitivity, assignment precision, and fitting degree, with the ISEM taste evaluation method proving to be the most effective. In order to further explore the bitterness superposition rules of different bitter substances (Zhang et al., 2021), selected nine types of Chinese medicinal slices as research carriers. On the basis of establishing a predictive model between the quality concentration of the monomer slice carrier and the bitterness tasted by mouth, they explored the relationship between the bitterness tasted by mouth when measuring the superposition of binary and ternary systems and the bitterness and quality concentration of the monomer slice. The research found that the quality concentration of the monomer slice can be well fitted to the predictive equation of the bitterness of the superimposed slices, and the contribution rate of Huanglian to the superimposed bitterness is often greater than that of the other components, fully confirming a Chinese saying, “A mute eats Huanglian, and the bitterness is unspeakable."

The bitterness determination method based on RB and SAB offers a direct approach to determining the bitterness of various substances, including monomeric compounds, decoction pieces, and compound decoctions. However, the measurement process may be influenced by the intrinsic structure, concentration, and temperature of the molecule. Therefore, controlling appropriate external conditions during the measurement process is essential.

#### 2.1.4 Ratio of bitterness (ROB)

In the sweetness evaluation method, there exists a calculation method known as “the relative sweetness value (RS)”, utilized for comparing different sweeteners. Researchers established the sweetness (Sr) of a 5% sucrose solution (*C*r) as one and determined the mass concentration of other sweet substances equivalent to their sweetness. The RS of the sweet compound was then calculated using the formula RSs = *C*r/*C*s × Sr (Park et al., 2017). Building upon this concept, researchers proposed a method for determining the “Ratio of Bitterness (ROB)” of bitter substances (Li et al., 2023). Specifically, they determined the specific bitterness (ROBr) of a BBR solution with a mass concentration of 0.05 mg/mL (*C*r) as one and obtained the mass concentration of other bitter substances equivalent to their bitterness. The ROB of the bitter compound was then calculated using the formula “ROBs = *C*r/*C*s × ROBr”. Due to the significant variance in their values, their natural logarithm is termed the ROB-index (ROBI). Serving as an absolute quantitative index, ROB reflects a fundamental attribute of bitter substances, facilitating a straightforward comparison of bitterness among different bitter substances. Following these principles, researchers successfully determined the ROB of six bitter drug monomers, offering a new bitterness scale for comparing bitterness across various bitter drug monomers and enhancing the scope of research on drug bitterness comparison scales (Li et al., 2023).

#### 2.1.5 Molecular bitterness (MB) and equivalent molecular bitterness (EMB)

Many drugs exhibit a bitter taste despite having different chemical structures. The emergence of bitterness is linked to factors such as the shape, size, and properties of functional groups within the molecule, as well as their positions. Eitan Margulis *et al* (Margulis et al., 2021) successfully constructed a machine learning tool, termed “BitterIntense,” based on the chemical structural features of molecules. By calculating molecular descriptors, the tool classifies them into categories of “very bitter” or “not very bitter” with an accuracy rate of over 80%. This is significant for the early stages of drug development, as it allows for the rapid identification of compounds with intense bitterness. However, this method is a simple binary classification of bitterness intensity. How to establish a more precise bitterness intensity prediction algorithm based on molecular structural features remains a question that scholars are currently exploring. Liu et al., 2012 addressed the influence of concentration on the bitterness of bitter substances and introduced the concept of “molecular bitterness,” which pertains solely to the properties of drug molecules. The bitterness threshold concentration (BTC) of both the standard bitterness substance and the compound under examination was determined using the “minimum limit method,” representing the lowest concentration at which bitterness is detected by half of the volunteers. The Molecular Bitterness (MB) under the standard bitterness substance BTC was set as 1 (typically using berberine hydrochloride as the reference bitter substance, with an MB of one under BTC). Calculating the MB of the test compounds involved the formula “MBs = *C*r/*C*s × MBr” (where *C*r signifies the BTC of the standard bitter molecule; MBr denotes the MB of standard bitter molecules; *C*s represents the BTC of unknown bitter molecules; MBs represents the MB of unknown bitter molecules (Heath et al., 2006; Bora et al., 2008; Li Xuelin et al., 2013; Jelvehgari et al., 2014; Gao et al., 2022)). Given the substantial variance in BTC among different bitter molecules, MB values differ significantly across substances. Therefore, the introduction of the “molecular bitterness index” (MB-Index, MBI) involves taking the natural logarithm of MB to normalize the magnitude difference, facilitating a more straightforward comparison of bitterness across different substances. Using this method, (Gao et al., 2022) calculated the MB of 19 bitter monomer components such as quinine (alkaloids), naringin (glycosides), andrographolide (terpenes), and L-arginine (peptides) to be 0.8398, 0.0551, 0.0058, and 0.0002, respectively. The corresponding MBI values were −0.1746, −2.8982, −5.1447, and −8.3669, respectively, effectively illustrating the bitterness characteristics of various bitter components in a simple and intuitive manner.

The introduction and application of the MB concept addressed the comparison of bitterness between compounds. However, for natural medicine decoction pieces, and even compounds composed of multiple natural medicine decoction pieces, the evaluation extends beyond a single compound to encompass the combination of various bitter compounds. The change in bitterness value within such complex systems after combination is intricate. With numerous types of natural medicines, there’s an urgent need to establish an objective and appropriate bitterness evaluation method. Taking bitter natural medicines as an example, the current 2020 edition of the “Chinese Pharmacopoeia” includes a total of 2,711 natural medicines. Among them, 133 natural medicines exhibit a single bitter taste, comprising one very bitter, 14 extremely bitter, 47 bitter, one slightly bitter, and 70 slightly bitter (Supplementary Table S1). Additionally, 180 natural medicines possess not only a bitter taste but also other flavors (Supplementary Table S2). There are also nuanced differences in the taste descriptions of various natural medicines; for instance, Gentiana is described as “very bitter,” Sophorae Tonkinensis as “extremely bitter,” Bletilla as “bitter,” Eucommia as “slightly bitter,” and Lily bulb as “a little bitter” However, the distinction in bitterness between each description remains unknown. Furthermore, natural drugs described as ‘extremely bitter,’ such as Sophora flavescens and Aloe vera, pose the question: is their bitterness identical? Drawing from the MB calculation principle and bitterness measurement, Liu *et al.* (Gao et al., 2022) introduced the concept of “Equivalent Molecular Bitterness (EMB)” for complex systems. This involves determining the BTC of standard bitter substances and unknown complex systems using the “minimum limit method.” The MBr under the standard bitter substance BTC is defined as 1 (typically using berberine hydrochloride as the reference bitter substance, with an MBr of one under BTC). Subsequently, the EMB calculation formula for other bitter Chinese herbal decoction pieces is “EMBs = *C*r/*C*s × MBr,” where *C*r represents the BTC of berberine hydrochloride, and *C*s denotes the BTC of unknown Chinese herbal pieces. The natural logarithm of this ratio is termed the EMB-index (EMBI) (Wang et al., 2022). measured the EMB and EMBI of 23 kinds of bitter Chinese herbal pieces using the aforementioned methods and established a quantitative method for determining the bitterness of bitter Chinese herbal pieces. This comparative analysis of the bitterness characteristics of different types of Chinese herbal pieces offers valuable insights, laying a robust foundation for the accurate masking of natural drugs within complex systems.

The comprehensive review reveals that researchers approach quantitative analysis of bitterness from diverse perspectives and levels using THTPM as a foundation. Each method presents distinct advantages, limitations, and applicability (Table 2). When embarking on quantitative investigations into bitterness, it is crucial to select appropriate methodologies tailored to the study’s objectives and the nature of the research subject.

**TABLE 2:** Analysis of the characteristics of various evaluation indexes of bitterness based on THTPM measurements.

Name	Quantitative description analysis	gLMS score	Rank bitterness intensity	Standard apparent bitterness	Ratio of bitterness	Molecular bitterness	Equivalent molecular bitterness
Abbreviation	QDA	gLMS	RB	SAB	ROB	MB/MBI (Index)	EMB/EMBI (Index)
Reference	Each has different	General Psychophysical Scale	Five specific concentrations of BBR (across low/medium/high bitterness)	Five specific concentrations of BBR (across low/medium/high bitterness)	0.05 mg · mL-1 BBR (SAB = 3, medium bitterness)	Bitterness threshold concentration (9.947 × 10^−6^ M/L, very low bitterness) of BBR	Bitterness threshold concentration (9.947 × 10^−6^ M/L, very low bitterness) of BBR
Relationship with concentration	Related (Logarithm/Weibull)	Related (Logarithm/Weibull)	Related (Logarithm/Weibull)	Related (Logarithm/Weibull)	Independence	Independence	Independence
Data characteristics	Relative value	Absolute value	Absolute value	Relative value	Absolute value	Absolute value	Absolute value
Advantages	Most of the bitter substance solution can be measured; suitable for monomer, decoction pieces, and compound decoctions	The evaluation process is simple, and suitability is the same as “QDA”	The same as “QDA”	The same as “QDA”	It has nothing to do with the concentration; it can be directly used to compare the intensity of bitterness; suitable for bitter monomer compounds	The same as “ROB”	It has nothing to do with the concentration; it can be directly used to compare the intensity of bitterness; it is suitable for the bitterness of decoction pieces, and compound decoctions
Disadvantages	Affected by the evaluator’s own factors; affected by factors such as the concentration of bitter substances and the determination temperature; the test methods of each research institution are different; it is difficult to make a direct horizontal comparison of the values due to the different participating carriers	Affected by the evaluator’s own factors; affected by the concentration of bitter substances, determination temperature and other factors; the test methods of each research institution are different	Affected by the evaluator’s own factors; affected by factors such as the concentration of bitter substances and the determination temperature; the test methods of each research institution are different; it is difficult to make a direct horizontal comparison of the values due to the different participating carriers	The same as “RB”	It is not suitable for the evaluation of low bitterness level at high concentration	Applicable to the bitterness evaluation of bitter monomers; bitterness prediction suitable for low bitterness of multiple monomers	Applicable to the bitterness evaluation of bitter decoction pieces; it is suitable for the bitterness prediction of low bitterness aqueous solutions of compound decoction pieces
Applicable conditions	It is widely used to evaluate the bitterness of most bitter substance solutions	It is widely used to evaluate the bitterness of most bitter substance solutions	Bitterness evaluation of most bitter substance solutions; prediction of bitterness under different concentrations of the same carrier	Bitterness evaluation of most bitter substance solutions; prediction of bitterness under different concentrations of the same carrier; it can directly compare and predict the low, medium, and high bitterness of the carrier; prediction of bitterness under different concentrations of the same carrier	Evaluation and comparison of ROB bitterness of bitter monomer compounds; bitterness prediction of medium bitterness aqueous solutions	Evaluation and comparison of MB bitterness of bitter monomer compounds; bitterness prediction of low bitterness solutions	Evaluation and comparison of MB bitterness of bitter decoction pieces; bitterness prediction of low bitterness compound solutions

### 2.2 ETM

**FIGURE 3:**
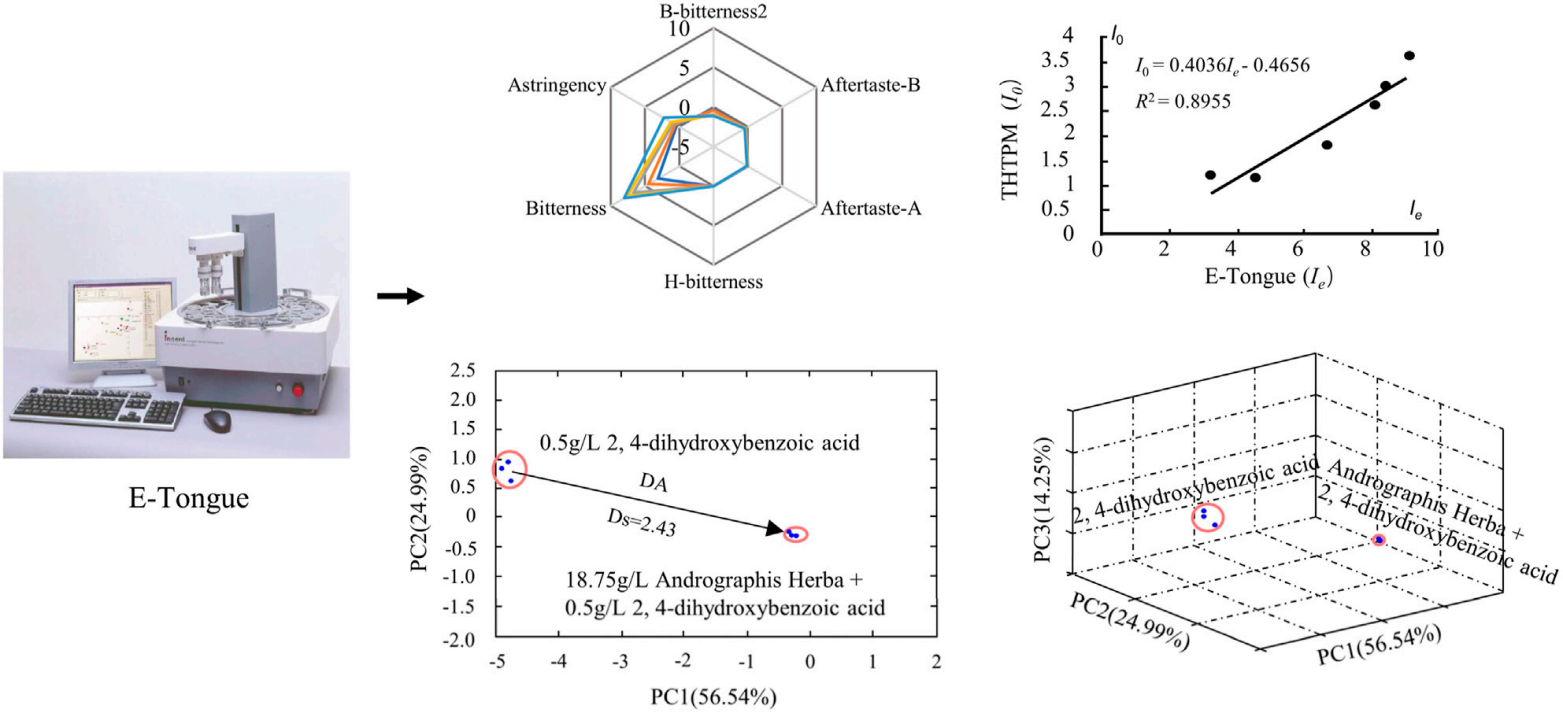
Quantitative research method of bitterness based on the ETM. Representative images were our own work (Rui-xin et al., 2013; Li et al., 2016; Gao et al., 2022).

Sensory group evaluation poses significant challenges due to medical ethics considerations, associated health risks, and the substantial costs of personnel training. Moreover, the inherent subjectivity among individuals can lead to fatigue, slow evaluation speeds, and a limited sample size. Throughout the evaluation process, there is a risk of sample perception migration and perception saturation, thereby imposing constraints on the assessment of taste within the general population (Legin et al., 2004). The Gustation Analytical Fingerprint Technique (GFAT) represents a recent development in taste recognition and detection technology, relying on taste sensors and chemical information processing methods. These taste sensors function as intelligent recognition electronic systems, emulating the human taste mechanism to generate signals (optical, electrochemical, electrophysiological). They possess the capability to discern subtle differences in basic tastes, such as lingering or transient tastes. Notably, GFAT offers advantages such as rapid analysis, low cost, minimal sample preparation, and automation of analysis (Rudnitskaya et al., 2010; Podrażka et al., 2017). Scholars have conducted a systematic evaluation of the application of ETM and sensory groups in taste assessments of pediatric drugs. The findings reveal that sensory tests for children are infrequent (10.3%), with ETM predominating in pediatric drug taste evaluations (57.5%), highlighting the efficacy of ETM (Guedes et al., 2021). Over the past few decades, leveraging electronic tongue technology, researchers have successfully employed methods such as the conversion of electronic tongue taste information value (Zeng et al., 2015; Li et al., 2016), bitter distance calculation, and the establishment of relationships between electronic tongue information and human sensory evaluation (Ito et al., 1998; Uchida et al., 2001). These advancements have facilitated the quantitative analysis and prediction of various drug tastes, as shown in Figure 3.

#### 2.2.1 Electronic tongue converts taste information value

Using the TS-5000Z multi-channel taste sensor as an illustration, it employs an artificial lipid membrane sensor technology akin to the functioning principle of human tongue taste cells. This sensor has the capability to objectively and digitally detect five basic tastes as well as flavor attributes such as “sharpness” and “richness.” In the TS-5000Z taste analysis system, Relative value (R) and Change of Membrane Potential caused by Adsorption (CPA) are commonly utilized (Li et al., 2020). To initiate taste analysis, the taste sensor is immersed in a reference solution comprising a mixture of KCl and tartaric acid at a predetermined concentration, yielding the corresponding membrane potential, denoted as Vr. This reference solution is essentially tasteless. Subsequently, the sensor is submerged in the sample solution to determine the potential difference value of the solution potential (Vs.), which is then subtracted from Vr, termed as the first taste (R). Following a gentle cleanse of the taste sensor with the reference solution, it is re-immersed to detect the potential Vr’. The disparity between Vr’ and Vr is referred to as aftertaste (CPA), indicating the potential change induced by chemical adsorption.

The first taste and aftertaste can be calculated by Equations 1, 2:

R=Vs‐Vr (1)

CPA=Vr′‐Vr (2)

Based on the initial taste and aftertaste values, a specific mathematical conversion is performed to derive the electronic tongue conversion taste information value (I.e.,), serving as a metric for quantitative bitterness evaluation (Zeng et al., 2015). employed the electronic tongue technology-based taste analysis method to quantify the characteristics of Scutellaria baicalensis from various sources. By establishing a positive correlation between the bitterness, astringency, bitter aftertaste, astringency aftertaste, sour taste information of Scutellaria baicalensis, and the baicalin content, it was possible to infer the baicalin content in Scutellaria baicalensis. In a similar vein (Jing et al., 2022), utilized electronic tongue technology to quantify the taste of 20 batches of Magnolia officinalis, assessing the taste of six monomer compounds. Pearson correlation analysis was employed to ascertain the correlation between eight chemical components and the taste sensor response value. The investigation revealed a significant positive correlation between honokiol, magnolol, and spicy menthol magnolol in Magnolia officinalis, and the bitter taste and bitter aftertaste detected by the electronic tongue.

#### 2.2.2 Distance of bitterness

##### 2.2.2.1 Distance of bitterness in multidimensional space

Using the French ASTREE electronic tongue method as a case study, the quantification of bitterness index relies on data gathered from seven sensors within the electronic tongue apparatus. Through meticulous data processing, a numerical value is derived, providing a quantitative or semi-quantitative representation of the drug’s bitterness. This value is termed the Bitterness Distance (D). Utilizing chemometric techniques such as PCA, the spatial disparity between the sample under examination and a reference solution is computed. This approach mirrors principles found in cluster analysis and other methodologies, where the distance between samples is evaluated within a multidimensional space comprising various variables.

Distance serves as a metric to gauge the spatial separation between two entities. Common distance metrics encompass Euclidean distance, Mahalanobis distance, Ming’s distance, among others. The Euclidean distance (EUCLID), or Euclidean metric, stands out as a prevalent distance measure, delineating the true geometric distance between two points within an m-dimensional space. Its applicability is underscored by its capacity to be expressed in a unified recursive formula, making it the most frequently utilized distance metric.

The Euclid and Standardized Euclid can be calculated by Equations 3, 4:

$$Euclid=\sqrt{\sum_{i=1}^{k}{\left(x_{i}‐y_{i}\right)}^{2}}$$ (3)

$$Standardized\;Euclid=\sqrt{\sum_{i=1}^{k}{\left(\frac{x_{i}‐y_{i}}{S_{i}}\right)}^{2}}$$ (4)

Where k represents the number of variables each sample possesses, with x_i_ indicating the value of the first sample on the i-th variable, and y_i_ representing the value of the second sample on the same variable. In the context of bitter samples, the Euclidean distance between samples exhibiting varying degrees of bitterness serves as a measure of the disparity in bitterness levels.

For instance, consider the compound BBR, which was formulated into samples of varying concentrations. Each sample, along with purified water, underwent analysis using an electronic tongue. The resulting dataset facilitated the direct calculation of its Euclidean distance, effectively quantifying the multidimensional space between them. Notably, a larger Euclidean distance between the sample and purified water signifies a higher bitterness level in the sample, and conversely, a smaller distance indicates lower bitterness.

##### 2.2.2.2 Distance of bitterness in reduced-dimensional space

The data collected by the electronic tongue underwent reduction via Principal Component Analysis (PCA) and similar techniques. Subsequently, based on these findings, the distance between each sample and the reference solution within the principal component space (whether in two-dimensional, three-dimensional, or other dimensions) was computed to determine the relative bitterness of each sample.

In a two-dimensional or three-dimensional space, the Euclidean distance serves as the measure of separation between two points, delineating the extent of spatial disparity, as shown in Equations 5, 6:

$${EUCLID}_{\left(2D\right)}=\sqrt{{\left(x_{1}‐x_{2}\right)}^{2}+{\left(y_{1}‐y_{2}\right)}^{2}}$$ (5)

$${EUCLID}_{\left(3D\right)}=\sqrt{{\left(x_{1}‐x_{2}\right)}^{2}+{\left(y_{1}‐y_{2}\right)}^{2}+{\left(z_{1}‐z_{2}\right)}^{2}}$$ (6)

When employing PCA for dimensionality reduction analysis, we can compare the bitterness differences among samples by assessing the distance from each sample to the reference solution in both two-dimensional and three-dimensional spaces. Nakamura *et al.* conducted a study to assess the taste of orally disintegrating tablets (ODT) containing famotidine and amlodipine besylate using the Astree electronic tongue and THTPM. The palatability of the tablets was further evaluated using a 100 mm VAS scale. The findings indicated that both physical masking and organoleptic masking could enhance the palatability of famotidine and amlodipine. In the electronic tongue analysis, the Euclidean distance of samples subjected to physical masking, organoleptic masking alone, and in combination, was found to be smaller compared to unmasked drugs (Nakamura et al., 2015). Liu *et al.* investigated bitter drug carriers employing BBR and Andrographis paniculata decoction, screening taste masking agents by assessing bitterness reduction values in reduced-dimensional or multi-dimensional space (Liu et al., 2013; Rui-xin et al., 2013). Li et al. (2011) assessed the masking effect of various agents on berberine hydrochloride using bitterness distance, D, and bitterness reduction distance, ΔD (Li et al., 2013).

While the results derived from PCA analysis slightly underperform compared to multi-dimensional space distance, they offer a more intuitive representation through two-dimensional or three-dimensional maps, overcoming the graphical limitations of multi-dimensional spaces. Moreover, data standardization aids in further reducing system errors. However, it is important to note that this method is only applicable to distinguishing bitterness within the same component.

#### 2.2.3 The relationship between electronic tongue taste information value and THTPM

The electronic tongue taste information is typically expressed through relative response values or bitterness values. Establishing the relationship between electronic tongue taste information and THTPM involves data-driven modeling and prediction, relying on experimental data and mathematical methods. Several studies have demonstrated a strong correlation between taste assessed by electronic tongue and human taste perception (Ito et al., 2013; Wang et al., 2013; Maniruzzaman et al., 2014; Maniruzzaman and Douroumis, 2015). In recent years, there has been a proliferation of applications for quantitatively predicting bitterness using electronic tongue. For instance, Li (Li et al., 2016) utilized berberine hydrochloride as a reference and matrine and oxymatrine as model drugs to establish a bitterness prediction model (BPM) based on THTPM bitterness ratings and data from the TS-5000Z electronic tongue sensor. The results indicated a significant correlation between taste bitterness and electronic tongue bitterness (R^2^_matrine_ = 0.8955, R^2^_oxymatrine_ = 0.9793). The electronic tongue-based bitterness prediction model for matrine and oxymatrine exhibited high accuracy (R^2^_matrine_ = 0.9639, R^2^_oxymatrine_ = 0.9535). (Liu et al., 2014a) developed a BPM for berberine hydrochloride using a genetic algorithm-back propagation neural network (GA-BP), incorporating bitterness intensity evaluated by sensory groups and data provided by electronic tongue. The model demonstrated excellent fitting (R^2^ = 0.99965) and could effectively predict the bitterness of berberine hydrochloride across different concentrations, serving as a reference for developing BPMs for other drugs. Chen (Chen et al., 2020) presented a biosensor utilizing *Drosophila* odorant binding protein (OBP) as a biosensitive material. This biosensor was employed to study typical bitter molecules such as denatonium, quinine, and berberine using electrochemical impedance spectroscopy. The findings revealed significant binding properties between the bitter molecules and OBP, displaying a linear response within the concentration range of 10-9-10–6 mg/mL, indicating broad application prospects for the OBP-based biosensor. (Xue, 2022) employed Weibull curve fitting to evaluate the taste of oseltamivir phosphate and ginkgo leaves, along with electronic tongue data, enabling quantitative description of bitterness. The prediction model’s accuracy and superiority were assessed through cross-validation. Additionally, the electronic tongue method’s ability to predict the bitterness of bitter substances was validated against THTPM results.

In general, there exists a certain correlation between the taste information provided by the electronic tongue and the outcomes from THTPM, although this correlation may not always be consistent. Numerous factors contribute to this, including the type of electronic tongue, sensor selection, signal processing methods, data analysis techniques, standardization of tasting methods, and the training of evaluators. Due to variations in perception mechanisms and sensitivity between electronic tongues and human taste, the electronic tongue may not fully capture the nuanced characteristics of individual taste perception (Uchida et al., 2001). Consequently, the relationship between electronic tongue taste information and taste assessment methods requires calibration and validation specific to the samples and conditions at hand and cannot be generalized.

### 2.3 Taste strips and filter paper disc method

**FIGURE 4:**
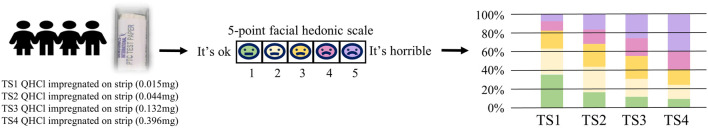
Quantitative research method of bitterness based on taste strips (TS). Based on (Ranmal et al., 2023).

Taste strips (TS) consist of filter paper infused with taste substances. When evaluating, the evaluator places the TS on the tongue’s center, closes the mouth, and gradually moves the tongue, allowing saliva to dissolve the taste enhancer on the strip. After a designated period, the strip is removed for taste assessment, as shown in Figure 4. Ranmal (Ranmal et al., 2023) examined subjects’ hedonistic responses to bitter stimuli from TS. Findings revealed that as the concentration of quinine hydrochloride (QHCl) on TS increased, both children and adults showed heightened aversion to bitterness. Similarly, Schienle (Schienle and Schlintl, 2020) utilized QHCl TS to gauge taste intensity, ranging from no sensation to “the strongest imaginable sensation of any kind.” Green (Green et al., 2022) employed TS containing high and low concentrations of four tastes (sour, sweet, bitter, and salty) to assess taste function in healthy participants. Results indicated elevated recognition levels among participants exposed to high-concentration taste strips in laboratory settings.

Another bitterness measurement method akin to the TS method is the filter paper disc method (FPD). KATARINA(Berling et al., 2011) employed FPD to assess evaluators’ perception thresholds for various flavors. Each flavor agent comprised five different concentrations. Using a scoring system from one to 6, where one indicates the lowest threshold, five represents the highest measurable threshold, and six signifies an unmeasurable high threshold, evaluators progressed from low to high concentrations until they correctly identified the taste, thus determining the recognition threshold. Results indicated standard thresholds for four flavors: bitter 1.9 ± 1.30, acid 2.3 ± 1.09, salty 2.5 ± 1.53, and sweet 2.6 ± 1.37, respectively, with bitterness identified at a lower concentration than other flavors.

The TS and FPD methods offer a straightforward, rapid, safe, and effective out-of-laboratory (OOL) sensory evaluation approach for assessing bitterness perception. Nonetheless, further research is warranted to establish a stronger correlation between the “local stimulation” method and the “full mouth” method based on the classical population taste evaluation method.

### 2.4 Facial expression analysis

**FIGURE 5:**
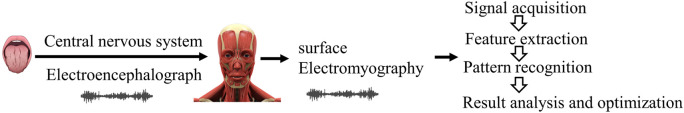
Quantitative research method of bitterness based on facial expressions (Wang, 2022a).

Facial expressions serve as a rich source of emotional information. When individuals taste different flavors of medications, their facial expressions vary accordingly. For instance, tasting non-bitter Chinese medicine may elicit “neutral” expressions, whereas tasting bitter Chinese medicine may provoke expressions of “disgust,” characterized by tight frowns and clenched teeth. Facial expression recognition technology leverages facial expression data to objectively analyze human emotional responses. Utilizing this technology, we can extract facial expression features of individuals and employ suitable expression classification methods to objectively assess taste perception, as shown in Figure 5.

Wang (2022a) utilized taste stimulation to perceive potential signals from nerve-related facial muscles and gland-related muscles, converting them into digital signals to acquire taste information, thus enabling the acquisition of taste information from electric potential signals. Furthermore, variations in the intensity of expression responses may occur when evaluators taste natural medicines with differing levels of bitterness (Zhi et al., 2017). observed that facial expression intensity can indicate the degree of taste stimulation across various concentrations and levels. Most participants displayed pronounced aversion to medium and high concentrations of bitterness, manifesting as expressions of disgust. With the rapid advancement of deep learning, facial expression recognition technology has progressed from simple classification to intensity level analysis. Yang et al., 2010 introduced a novel technique for facial expression analysis based on a ranking model. They transformed the task of expression intensity analysis into a ranking problem and employed RankBoost modeling. The resulting ranking score can directly estimate intensity and demonstrated good performance on the Cohn-Kanade dataset. As facial expression recognition technology continues to evolve, researchers have established datasets such as JAFFE, FER 2013, and CK + for facial expression analysis. However, an exclusive dataset for bitterness evaluation is yet to be established. Developing such a dataset is crucial to advancing the intelligent and accurate quantification of bitterness.

### 2.5 Animal behavior test

**FIGURE 6:**
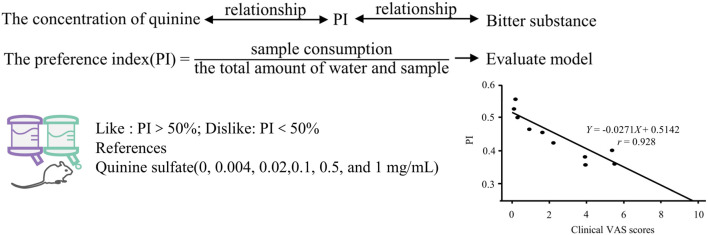
Quantitative research method of bitterness based on animal behavior test. Based on (Han et al., 2018).

When one animal is attracted to a stimulus while another avoids it, it suggests that the compound may possess distinct perceptual characteristics for different tasters, leading to varied evaluations (Loney et al., 2012). The two-bottle preference test (TBP) (Yoneda et al., 2007) is employed to assess the aversive taste of food or beverages, utilizing the preference index (PI) as the evaluation metric (Loney et al., 2011). Rodents are commonly chosen as experimental subjects due to their highly homologous bitter taste receptors to humans, thus exhibiting similar taste perceptions (Noorjahan et al., 2014). Han (Han et al., 2018) established the relationship between quinine concentration and animal PI. Subsequently, the PI of 12 bitter Traditional Chinese herbal (TCH) compounds was determined using TBP, and the bitterness results were standardized into a unified numerical system based on the concentration-PI relationship. This standardization offers a methodological framework for sensory evaluation of natural medicines, as shown in Figure 6.

Magdalena Münster (Münster et al., 2017) assessed the palatability of the bitter compound praziquantel using the rodent transient contact taste aversion (BATA) model and calculated the IC_50_ value, representing the concentration of praziquantel inhibiting 50% of the maximum licking response. The findings revealed a decrease in licking frequency with increasing praziquantel concentration, with an IC_50_ value of 0.06 mg/mL (95% CI 0.049-0.082). Comparative analysis indicated that praziquantel elicited a stronger aversive response compared to other bitter compounds such as sildenafil citrate, caffeine citrate, diclofenac, or paracetamol (Soto, 2016).

It is important to note that the outcomes of animal studies are influenced by species-specific expression of bitter taste receptors, resulting in bitter taste responses that may differ from those in humans (Dong et al., 2009). Future research endeavors should focus on refining methodologies to achieve more accurate quantitative assessments of bitter taste.

### 2.6 Cell-based assessment methods

**FIGURE 7:**
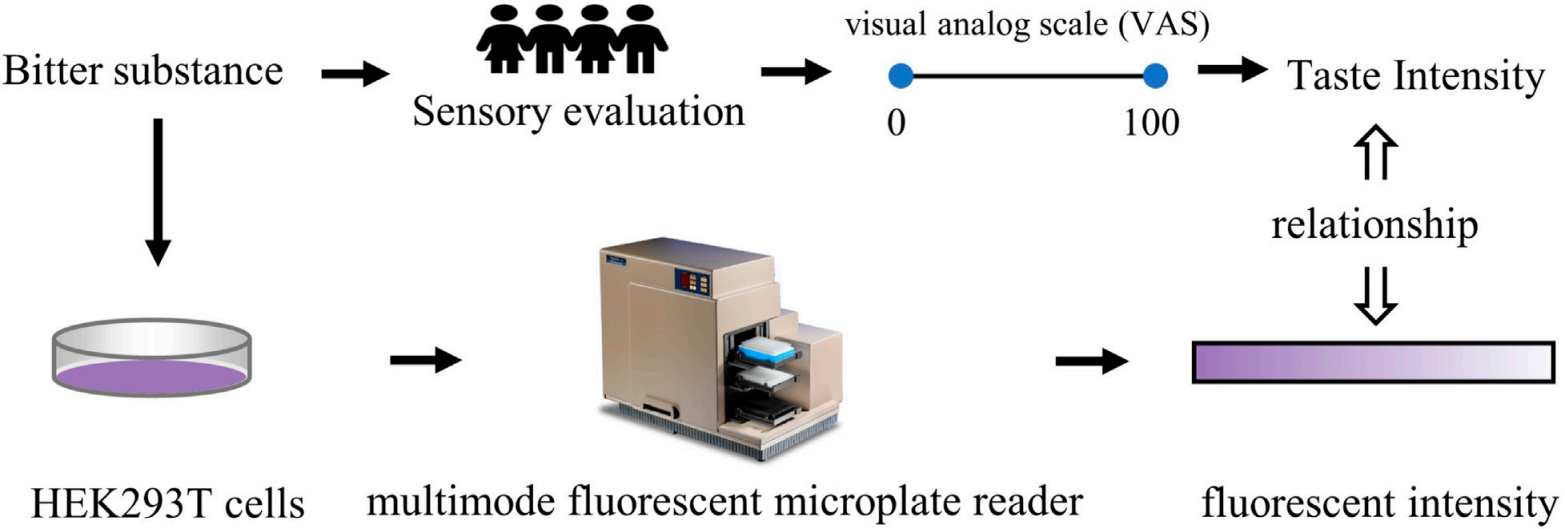
Quantitative research method of bitterness based on animal behavior test. Based on (Narukawa et al., 2011).

Bitter substances, serving as flavoring agents, can stimulate certain taste bud cells. By describing the interaction strength between them, it is possible to achieve an objective measurement for the quantification of bitterness (Narukawa et al., 2011), as shown in Figure 7. (Hui et al., 2012) utilized human intestinal endocrine STC-1 cells expressing G protein-coupled receptors and bitter receptors (type 2 members) as sensing devices to conduct specific detection of bitter substances. The findings demonstrated that the sensor utilizing STC-1 cells selectively responded to bitter agents and mixtures, with the type and concentration of bitter agents determinable via signal-to-noise ratio parameters. This approach offers a valuable avenue for investigating taste mechanisms and evaluating bitterness intensity. Nakamura (Nakamura et al., 2003) investigated the effect of quinine on [[Ca^2 +^]i levels in cultured nerve-2a cells, exploring the potential of [[Ca^2 +^]i levels to predict the bitterness of quinine solutions. Following quinine stimulation, [Ca^2^+]i levels in nerve-2a cells increased in a concentration-dependent manner.

However, the cell-based biosensor evaluation method also has certain limitations, as cells may not be able to detect all bitter substances. For instance, Thomas (Delompré et al., 2022) demonstrated the bitterness of vitamins B2 and B3 in sensory analysis, where cell-based assays failed to yield any information. This may be attributed to the inherent fluorescence characteristics of the two vitamins at high concentrations (Chen and Chung, 2022). Additionally, current taste cell culture methods are susceptible to the influence of pseudo-taste cells, potentially leading to overinterpretation. Therefore, caution must be exercised when employing this method.

In summary, researchers have conducted numerous quantitative studies on bitterness using both *in vivo* and *in vitro* methods. Throughout this process, researchers have identified various dimensions of bitterness quantification, including local and overall characteristics, static and dynamic features, and external macro performance and internal micro mechanisms. Each method possesses its own advantages and disadvantages (Table 3). When evaluating drug development, taste masking, and palatability, researchers can select appropriate methods based on research objectives, cost, time constraints, and other factors. However, it is important to note that bitterness research methods are still evolving. In the future, researchers need to continue exploring quantitative evaluation methods for bitterness, standardizing the evaluation process to facilitate the high-quality development of bitterness quantification.

**TABLE 3:** Analysis of the advantages and disadvantages of various bitterness quantitative methods.

Name	THTPM	Taste strips and filter paper disc method	ETM	Facial expression analysis	Animal behavior test	Cell-based assessment methods
Advantages	It can intuitively give the taste of bitter substances	It can intuitively give the taste of bitter substances	It can objectively collect the taste data information of bitter substances; it is easy to operate, has low cost, has high sensitivity, and has a low detection threshold; it exhibits good repeatability and does not need ethical approval	It can be used for people who cannot express their evaluations through language	The taste of the sample can be judged by the number of animal intakes of the experimental sample or some escape reactions, such as shaking of the head or agitation; results are relatively intuitive	It is helpful for understanding the mechanism and characteristics of bitter substances
Disadvantages	Strong subjectivity, easy fatigue, poor repeatability, and limitations for toxic substances; the requirements for taste sensitivity of the evaluators are high; it is time-consuming and laborious; the number of samples for a single test is small and the operation is cumbersome, so it is not suitable for rapid taste evaluation of a large number of samples	The bitterness characteristics obtained by “local stimulation” are limited	The results are not intuitive enough, and further processing and analysis is needed through model algorithms; the sensor richness and detection limits need to be improved	It is susceptible to factors such as occlusion, illumination, and local changes in subtle expressions	The properties of the test samples cannot be obtained; can only be used as a supplement to the taste evaluation of the population; generally applicable for comparisons of bitterness intensity between compounds with known bitterness	Limited access to information; easy to be affected by the fluorescent cells; pseudo-flavor cells, resulting in negative results or over-interpretation

## 3 Future research directions

**FIGURE 8:**
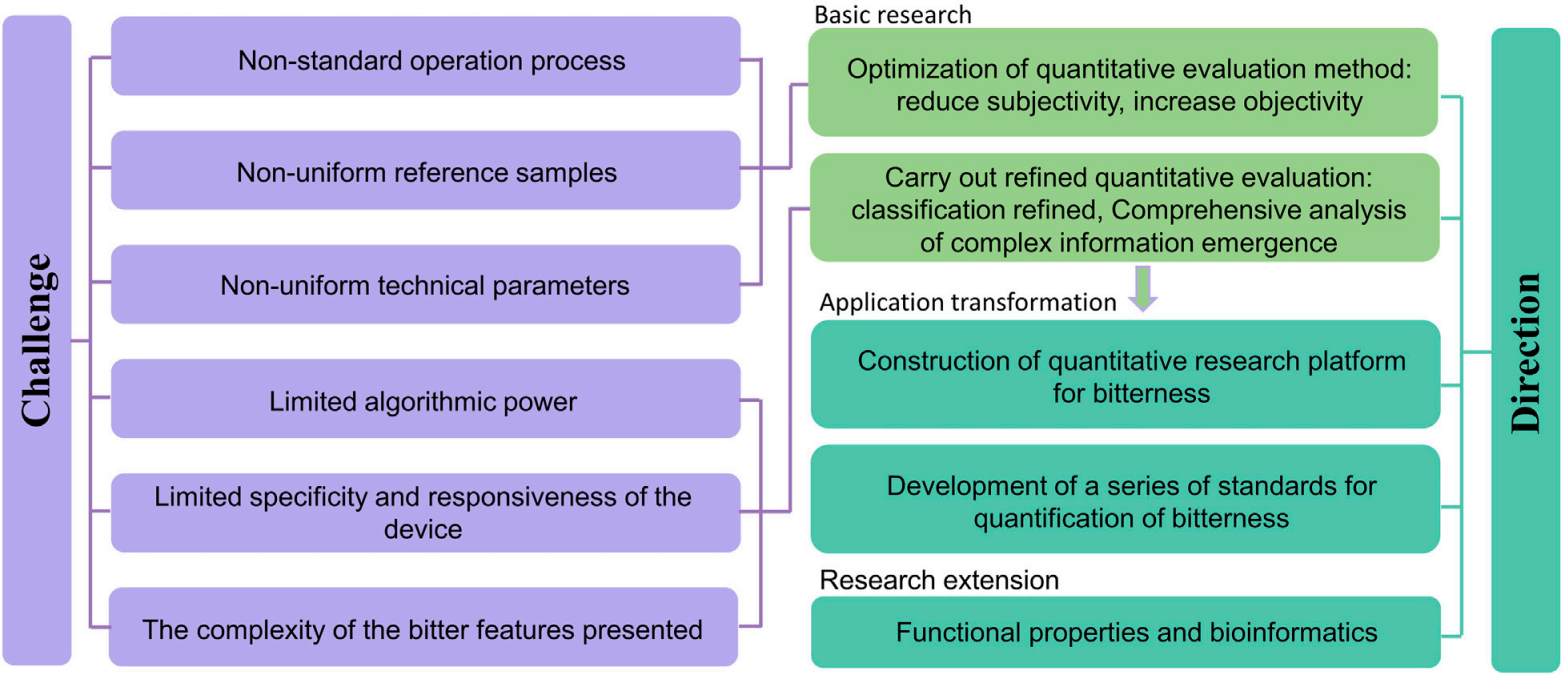
A framework addressing the challenges and future development directions in bitterness quantification.

Due to the influence of ethical reviews, the complexity of the regularity of bitter substance structural characteristics, the significant differences in the activation capacity of bitter taste receptors, and the surrounding environment of bitter substances, the quantitative research methods for bitterness are still actively being explored. The current research method system for bitterness, which is primarily based on THTPM and supplemented by other research methods, still requires further improvement to meet basic research needs. The main aspects which is shown in Figure 8 include: (1) Optimizing the quantification and evaluation methods for bitterness. By strictly selecting the evaluation population, establishing standardized operating procedures, and developing methods for handling outliers, the subjectivity of direct bitterness evaluation methods is reduced (Medicine, 2024); by strengthening the basic research on the structural characteristics of bitter substances, the characteristics of activating ligands, and the mechanisms of bitterness presentation, the relationship between concentration-structure-function-bitterness is explored, as well as the relationship between key chromatographic information/electrical signals/fluorescent signals and bitterness. This provides foundational support for optimizing indirect bitterness evaluation methods and actively utilizes machine learning algorithms to enhance the objectivity, accuracy, speed, and transparency of indirect bitterness evaluation methods. (2) Conduct refined quantification of bitterness and explore new methods for bitterness quantification research. Since there is a subtle relationship between people’s preferences or aversions to bitterness (Mura et al., 2018), research methods from the food field can be referenced to make refined distinctions in bitterness, such as good bitterness and bad bitterness, and to carry out refined quantitative evaluation of different types of bitterness in drugs (Sook Chung and Lee, 2012; Araujo et al., 2021). At the same time, closely focus on the taste-affecting factors that influence the bitterness of drugs and construct new methods for bitterness quantification and evaluation. For example, based on methods such as virtual screening, biofishing, and physicochemical detection, establish the relationship between key parameters of the above methods and bitterness, and systematically analyze the comprehensive impact of structural characteristics, concentration, and external environmental factors of bitter substances on bitterness. (3) Construct a quantitative research platform for bitterness. Currently, researchers often reveal the mechanisms of bitterness from a mesoscopic or microscopic perspective, and the bitterness platforms constructed are mostly centered around qualitative identification (determining whether it is bitter or not) (Chu et al., 2024). On this basis, there is an urgent need to build a quantitative research platform and equipment for bitterness, and to integrate different types of data in multiple dimensions, to promote the transformation of basic research on bitterness quantification to applied research. (4) Improve and establish a series of standards for quantitative research on bitterness. In order to achieve scientific measurement and effective evaluation of senses, a series of international documents have been issued for sensory analysis. For the sensory evaluation of bitterness, some scholars have already conducted research on the technical specifications for sensory evaluation based on the characteristics of natural medicines (Medicine, 2024). In the future, it is still necessary to formulate industry, national, and global standards around the research design and plan framework guidelines, statistical analysis plans, methodological validation, data processing, etc., of bitterness quantification, to promote the standardization, scientification, and systematization of quantitative research on drug bitterness. (5) Research Extension. Bitter substances possess a variety of physiological activities (Zuluaga, 2024). In traditional Chinese medicine theory, bitterness is believed to have effects such as " downbearing and discharging, drying dampness, and consolidating Yin " Advancing the research on the functional attributes of bitterness and its extension into the field of bioinformatics, including the relationships between bitterness and efficacy, and bitterness and receptors, can provide support for accelerating the development of target drugs.

In the process of exploring the quantification of bitterness, we also face many challenges. On one hand, there are issues such as non-standardized operating procedures and inconsistent technical parameters. These mainly include the lack of uniformity in scales, reference solutions, the volume of samples evaluated at one time, temperature, evaluation time and intervals, and limited instrument stability, which hampers the comparability of results between different studies (Zuluaga, 2024). On the other hand, the complexity of bitter taste presentation makes the quantification of bitterness very difficult, especially for the measurement of the comprehensive bitterness in complex systems, where it is urgent to explore the taste rules in the independent state of substances and under the state of complex systems (Zhang et al., 2021; Gao et al., 2023). In addition, the flexibility of virtual screening methods, the specificity and sensitivity of indirect measurement methods, and the computational power of different machine algorithms also affect the accuracy of bitterness quantification. In the future, researchers urgently need to further enrich the database of bitter substances, establish standardized and unified operating standards, and by improving detection technology and optimizing algorithm capabilities, jointly explore and mutually verify the characteristics of bitterness from macroscopic, mesoscopic, or microscopic perspectives (Li et al., 2024).

## 4 Conclusion and foresight

In nature, various taste substances exist alongside intricate taste mechanisms, and numerous factors influence the quantification of drug bitterness to varying extents. In light of this, different quantitative evaluation methods for bitterness have been established, each possessing its own merits. Presently. Currently, an increasing number of researchers are leveraging column chromatography, HPLC, HPLC/ESI-MS, LC/ESI-MS/MS, UPLC-Q-TOF/MS, and nontargeted LC/MS flavoromics analysis to separate and identify the bitter compounds (Suryawanshi et al., 2006; Mustafa et al., 2015; Höhme et al., 2023). They also combine methods such as sensory-guided, virtual screening, and chromatography-taste association to improve the efficiency of discovering bitter components (Yu et al., 2020; Yang et al., 2023). This signifies that the study of bitterness in natural medicines is steadily advancing. However, the identification of bitter components represents merely the initial phase. A precise, dependable, and straightforward method for evaluating drug bitterness is required to investigate bitterness masking strategies for medications. Similar to the measurement of length using the international unit “meter” and temperature using the “degree Celsius,” bitterness should also be subject to standardized, objective quantitative methods and parameters. This review scrutinizes research on bitterness quantification, delineates factors influencing drug bitterness, and acknowledges the role of material, human, and environmental factors in affecting bitterness perception. Consequently, in the quantitative exploration of drug bitterness, it is imperative to identify and regulate these factors to ensure the reliability of outcomes. Furthermore, this paper consolidates the characteristics of various bitterness quantification methods, systematically categorizes the quantitative approaches for representative drugs, and emphasizes the challenges associated with quantifying bitterness in natural drugs characterized by complex systems. It also elucidates the future research directions that urgently need to be undertaken. This is of significant guiding importance for our continued in-depth focus on the research of quantitative bitterness methods and lays an important foundation for the development of precise, efficient, and rich taste-masking strategies. Such efforts aim to foster research into taste masking optimization and palatability enhancement, thereby laying a crucial groundwork for enhancing the clinical acceptance of natural medications.

## References

[B1] Aliani M. Eskin M. N. A. (2017). Bitterness: perception. Chem. Food Process., 239–243. 10.1002/9781118590263

[B2] Amin F. Khan S. Shah S. M. H. Rahim H. Hussain Z. Sohail M. (2018). A new strategy for taste masking of azithromycin antibiotic: development, characterization, and evaluation of azithromycin titanium nanohybrid for masking of bitter taste using physisorption and panel testing studies. Drug Des. Devel Ther. 12, 3855–3866. 10.2147/dddt.S183534 PMC6231451 30510401

[B3] Araujo L. D. Parr W. V. Grose C. Hedderley D. Masters O. Kilmartin P. A. (2021). In-mouth attributes driving perceived quality of Pinot noir wines: sensory and chemical characterisation. Food Res. Int. 149, 110665. 10.1016/j.foodres.2021.110665 34600667

[B4] Aree T. (2019). Understanding structures and thermodynamics of β-cyclodextrin encapsulation of chlorogenic, caffeic and quinic acids: implications for enriching antioxidant capacity and masking bitterness in coffee. Food Chem. 293, 550–560. 10.1016/j.foodchem.2019.04.084 31151647

[B5] Bahia M. S. Nissim I. Niv M. Y. (2018). Bitterness prediction in-silico: a step towards better drugs. Int. J. Pharm. 536 (2), 526–529. 10.1016/j.ijpharm.2017.03.076 28363856

[B6] Beauchamp G. K. (2016). Why do we like sweet taste: a bitter tale? Physiol. Behav. 164 (Pt B), 432–437. 10.1016/j.physbeh.2016.05.007 27174610 PMC5003684

[B7] Berling K. Knutsson J. Rosenblad A. von Unge M. (2011). Evaluation of electrogustometry and the filter paper disc method for taste assessment. Acta Otolaryngol. 131 (5), 488–493. 10.3109/00016489.2010.535850 21391775

[B8] Bishop L. R. (2013). EUROPEAN BREWERY CONVENTION THE E.B.C. SCALE OF BITTERNESS. J. Inst. Brew. 73 (6), 525–527. 10.1002/j.2050-0416.1967.tb03078.x

[B9] Boesveldt S. de Graaf K. (2017). The differential role of smell and taste for eating behavior. Perception 46 (3-4), 307–319. 10.1177/0301006616685576 28056650

[B10] Bohin M. C. Roland W. S. Gruppen H. Gouka R. J. van der Hijden H. T. Dekker P. (2013). Evaluation of the bitter-masking potential of food proteins for EGCG by a cell-based human bitter taste receptor assay and binding studies. J. Agric. Food Chem. 61 (42), 10010–10017. 10.1021/jf4030823 24093533

[B11] Bora D. Borude P. Bhise K. (2008). Taste masking by spray-drying technique. AAPS PharmSciTech 9 (4), 1159–1164. 10.1208/s12249-008-9154-5 19016332 PMC2628278

[B12] Chen Z. Chung H. Y. (2022). Pseudo-taste cells derived from rat taste and non-taste tissues: implications for cultured taste cell-based biosensors. J. Agric. Food Chem. 70 (35), 10826–10835. 10.1021/acs.jafc.2c04934 35998688

[B13] Chen Z. Zhang Q. Shan J. Lu Y. Liu Q. (2020). Detection of bitter taste molecules based on odorant-binding protein-modified screen-printed electrodes. ACS Omega 5 (42), 27536–27545. 10.1021/acsomega.0c04089 33134717 PMC7594143

[B14] Christensen J. Ladefoged A. M. Nrgaard L. (2005). Rapid determination of bitterness in beer using fluorescence spectroscopy and chemometrics. J. Inst. Brew. 111 (1), 3–10. 10.1002/j.2050-0416.2005.tb00642.x

[B15] Chu X. Zhu W. Li X. Su E. Wang J. (2024). Bitter flavors and bitter compounds in foods: identification, perception, and reduction techniques. Food Res. Int. 183, 114234. 10.1016/j.foodres.2024.114234 38760147

[B16] Clapham D. Belissa E. Inghelbrecht S. Pensé-Lhéritier A. M. Ruiz F. Sheehan L. (2023). A guide to best practice in sensory analysis of pharmaceutical formulations. Pharmaceutics 15 (9), 2319. 10.3390/pharmaceutics15092319 37765288 PMC10535428

[B17] Cruickshanks K. J. Schubert C. R. Snyder D. J. Bartoshuk L. M. Huang G. H. Klein B. E. (2009). Measuring taste impairment in epidemiologic studies: the beaver dam offspring study. Ann. N. Y. Acad. Sci. 1170, 543–552. 10.1111/j.1749-6632.2009.04103.x 19686191 PMC2729771

[B18] Dagan-Wiener A. Nissim I. Ben Abu N. Borgonovo G. Bassoli A. Niv M. Y. (2017). Bitter or not? BitterPredict, a tool for predicting taste from chemical structure. Rep 7 (1), 12074. 10.1038/s41598-017-12359-7 PMC5608695 28935887

[B19] Delompré T. Belloir C. Martin C. Salles C. Briand L. (2022). Detection of bitterness in vitamins is mediated by the activation of bitter taste receptors. Nutrients 14 (19), 4141. 10.3390/nu14194141 36235793 PMC9571608

[B20] Deng M. Hida N. Yamazaki T. Morishima R. Kato Y. Fujita Y. (2022). Comparison of bitterness intensity between prednisolone and quinine in a human sensory test indicated individual differences in bitter-taste perception. Pharmaceutics 14 (11), 2454. 10.3390/pharmaceutics14112454 36432645 PMC9693378

[B21] Dong D. Jones G. Zhang S. (2009). Dynamic evolution of bitter taste receptor genes in vertebrates. BMC Evol. Biol. 9, 12. 10.1186/1471-2148-9-12 19144204 PMC2646699

[B22] Donley J. R. (1992). Solid-phase extraction of hop acids from beer or wort for subsequent analysis. J. Am. Soc. Brew. Chem. 50 (3), 89–93.

[B23] Finger T. E. Danilova V. Barrows J. Bartel D. L. Vigers A. J. Stone L. (2005). ATP signaling is crucial for communication from taste buds to gustatory nerves. Science 310 (5753), 1495–1499. 10.1126/science.1118435 16322458

[B24] Gao C. Tello E. Peterson D. G. (2023). Identification of compounds that enhance bitterness of coffee brew. Food Chem. 415, 135674. 10.1016/j.foodchem.2023.135674 36868066

[B25] Gao X. Bai X. Gui X. Wang Y. Wang J. Yao J. (2022). Study on quantitative method of drug’s molecular bitterness based on bitterness threshold concentration. Chin. Traditional Herb. Drugs 53 (3), 8. 10.7501/j.issn.0253-2670.2022.03.007

[B26] Gaudette N. J. Pickering G. J. (2013). Modifying bitterness in functional food systems. Crit. Rev. Food Sci. Nutr. 53 (5), 464–481. 10.1080/10408398.2010.542511 23391014

[B27] Ginz M. Engelhardt U. H. (2000). Identification of proline-based diketopiperazines in roasted coffee. J. Agric. Food Chem. 48 (8), 3528–3532. 10.1021/jf991256v 10956144

[B28] Green B. G. Dalton P. Cowart B. Shaffer G. Rankin K. Higgins J. (1996). Evaluating the 'Labeled Magnitude Scale' for measuring sensations of taste and smell. Chem. Senses 21 (3), 323–334. 10.1093/chemse/21.3.323 8670711

[B29] Green B. G. Shaffer G. S. Gilmore M. M. (1993). Derivation and evaluation of a semantic scale of oral sensation magnitude with apparent ratio properties. Chem. Senses 18 (6), 683–702. 10.1093/chemse/18.6.683

[B30] Green T. Wolf A. Oleszkiewicz A. Aronis A. Hummel T. Pepino M. (2022). Subjective assessment and taste strips testing of gustatory function, at home, and in the lab. bioRxiv, 507407. 10.1101/2022.09.11.507407

[B31] Guedes M. D. V. Marques M. S. Guedes P. C. Contri R. V. Kulkamp Guerreiro I. C. (2021). The use of electronic tongue and sensory panel on taste evaluation of pediatric medicines: a systematic review. Pharm. Dev. Technol. 26 (2), 119–137. 10.1080/10837450.2020.1860088 33274664

[B32] Guinard J. X. Hong D. Y. Budwig C. (2010). TIME‐INTENSITY PROPERTIES OF SWEET AND BITTER STIMULI: IMPLICATIONS FOR SWEET AND BITTER TASTE CHEMORECEPTION. J. Sens. Stud. 10 (1), 45–71. 10.1111/j.1745-459x.1995.tb00004.x

[B33] Guinard J. X. Hong D. Y. Zoumas-Morse C. Budwig C. Russell G. F. (1994). Chemoreception and perception of the bitterness of isohumulones. Physiol. Behav. 56 (6), 1257–1263. 10.1016/0031-9384(94)90374-3 7878099

[B34] Guinard J. X. Pangborn R. M. Lewis M. J. (1986). Effect of repeated ingestion on temporal perception of bitterness in beer. J. Am. Soc. Brew. Chem. 44 (1), 28–32. 10.1094/asbcj-44-0028

[B35] Gunaratne T. M. Fuentes S. Gunaratne N. M. Torrico D. D. Gonzalez Viejo C. Dunshea F. R. (2019). Physiological responses to basic tastes for sensory evaluation of chocolate using biometric techniques. Foods 8 (7), 243. 10.3390/foods8070243 31284449 PMC6679144

[B36] Han X. Jiang H. Han L. Xiong X. He Y. Fu C. (2018). A novel quantified bitterness evaluation model for traditional Chinese herbs based on an animal ethology principle. Acta Pharm. Sin. B 8 (2), 209–217. 10.1016/j.apsb.2017.08.001 29719781 PMC5925219

[B121] Hans‐Dieter B. Herbert W. (1985). Bitter compounds: occurrence and structure activity relationships. Food Rev. Int. 2, 271–354. 10.1080/87559128509540773

[B37] Hayes J. E. Wallace M. R. Knopik V. S. Herbstman D. M. Bartoshuk L. M. Duffy V. B. (2011). Allelic variation in TAS2R bitter receptor genes associates with variation in sensations from and ingestive behaviors toward common bitter beverages in adults. Chem. Senses 36 (3), 311–319. 10.1093/chemse/bjq132 21163912 PMC3038275

[B38] Heath T. P. Melichar J. K. Nutt D. J. Donaldson L. F. (2006). Human taste thresholds are modulated by serotonin and noradrenaline. J. Neurosci. 26 (49), 12664–12671. 10.1523/JNEUROSCI.3459-06.2006 17151269 PMC6674841

[B40] Höhme L. Fischer C. Kleinschmidt T. (2023). Characterization of bitter peptides in casein hydrolysates using comprehensive two-dimensional liquid chromatography. Food Chem. 404 (Pt A), 134527. 10.1016/j.foodchem.2022.134527 36242962

[B41] Howard G. A. (1968). Institute of brewing analysis committee estimation of the bitterness of beer. J. Inst. Brew. 74 (3), 249–251. 10.1002/j.2050-0416.1968.tb03121.x

[B42] Hu S. Liu X. Zhang S. Quan D. (2023). An overview of taste-masking technologies: approaches, application, and assessment methods. AAPS PharmSciTech 24 (2), 67. 10.1208/s12249-023-02520-z 36788171

[B43] Hu X. Li Y. Zhang E. Wang X. Xing M. Wang Q. (2013). Preparation and evaluation of orally disintegrating tablets containing taste-masked microcapsules of berberine hydrochloride. AAPS PharmSciTech 14 (1), 29–37. 10.1208/s12249-012-9880-6 23180226 PMC3581648

[B44] Hui G. Mi S. Ye S. Jin J. Chen Q. Yu Z. (2014). Tastant quantitative analysis from complex mixtures using taste cell-based sensor and double-layered cascaded series stochastic resonance. Electrochimica Acta 136, 75–88. 10.1016/j.electacta.2014.05.060

[B45] Hui G. H. Mi S. S. Deng S. P. (2012). Sweet and bitter tastants specific detection by the taste cell-based sensor. Biosens. Bioelectron. 35 (1), 429–438. 10.1016/j.bios.2012.02.028 22424755

[B46] Immohr L. I. Dischinger A. Kühl P. Kletzl H. Sturm S. Günther A. (2017). Early pediatric formulation development with new chemical entities: opportunities of e-tongue besides human taste assessment. Int. J. Pharm. 530 (1-2), 201–212. 10.1016/j.ijpharm.2017.07.069 28750893

[B47] Ito M. Ikehama K. Yoshida K. Haraguchi T. Yoshida M. Wada K. (2013). Bitterness prediction of H1-antihistamines and prediction of masking effects of artificial sweeteners using an electronic tongue. Int. J. Pharm. 441 (1-2), 121–127. 10.1016/j.ijpharm.2012.11.047 23247016

[B48] Ito T. Radecka H. Tohda K. Odashima K. Umezawa Y. (1998). On the mechanism of unexpected potentiometric response to neutral phenols by liquid membranes based on quaternary ammonium SaltsSystematic experimental and theoretical approaches. J. Am. Chem. Soc. 120 (13), 3049–3059. 10.1021/ja973179v

[B49] Jelvehgari M. Barghi L. Barghi F. (2014). Preparation of chlorpheniramine maleate-loaded alginate/chitosan particulate systems by the ionic gelation method for taste masking. Jundishapur J. Nat. Pharm. Prod. 9 (1), 39–48. 10.17795/jjnpp-12530 24644438 PMC3957142

[B50] Jing W. Zhao X. Zhang Q. Cheng X. Ma S. Wei F. (2022). Material basis of“Bitterness”Medicinal properties of magnoliae officinalis cortex based on electronic tongue and multi-component quantitative technology. Mod. Chin. Med. 24 (002), 258. 264Xue, W. (2022). *Study on the taste masking of two bitter substances based on the combination of taste method and electronic tongue technology.* master.

[B51] Ke X. Ma H. Yang J. Qiu M. Wang J. Han L. (2022). New strategies for identifying and masking the bitter taste in traditional herbal medicines: the example of Huanglian Jiedu Decoction. Front. Pharmacol. 13, 843821. 10.3389/fphar.2022.843821 36060004 PMC9431955

[B52] Legin A. Rudnitskaya A. Clapham D. Seleznev B. Lord K. Vlasov Y. (2004). Electronic tongue for pharmaceutical analytics: quantification of tastes and masking effects. Anal. Bioanal. Chem. 380 (1), 36–45. 10.1007/s00216-004-2738-3 15365669

[B53] Lemon C. H. Norris J. E. Heldmann B. A. (2019). The TRPA1 ion channel contributes to sensory-guided avoidance of menthol in mice. eNeuro 6 (6), ENEURO.0304–19.2019. 10.1523/eneuro.0304-19.2019 PMC6825956 31624176

[B54] Li C. Ren Y. Yao J. Wang Y. Gao X. Han Li (2023). Study on quantitative method of specific bitterness of bitter compounds based on traditional human taste panel method. Chin. Traditional Herb. Drugs 54 (09), 2758–2764.

[B55] Li C. Ren Y. Yao J. Wang Y. Gao X. Li H. (2023). Study on quantitative method of specific bitterness of bitter compounds based on traditional human taste panel method. Chin. Traditional Herb. Drugs 54 (09), 2758–2764.

[B56] Li C. Yao J. Zhang P. Dai X. Hou F. Shi J. (2024). Application of computer simulation in the taste-masking of traditional Chinese medicine decoction. Her. Med. 43 (07), 1107–1111. 10.3870/j.issn.1004-0781.2024.07.015

[B57] Li H. H. Luo L. Y. Wang J. Fu D. H. Zeng L. (2019). Lexicon development and quantitative descriptive analysis of Hunan fuzhuan brick tea infusion. Food Res. Int. 120, 275–284. 10.1016/j.foodres.2019.02.047 31000240

[B58] Li S. Zhang Y. Khan A. R. He S. Wang Y. Xu J. (2020). Quantitative prediction of the bitterness of atomoxetine hydrochloride and taste-masked using hydroxypropyl-β-cyclodextrin: a biosensor evaluation and interaction study. Asian J. Pharm. Sci. 15 (4), 492–505. 10.1016/j.ajps.2019.11.001 32952672 PMC7486553

[B59] Li X. Gui X. Liu R. Gao X. Meng X. Chen P. (2016). Bitterness intensity prediction of bitter compounds of traditional Chinese medicine based on an electronic tongue. Chin. J. New Drugs 25 (11), 1307–1314.

[B60] Li X. Li H. Liu R. Zhang X. Qiu J. Wu Z. (2013). Study on the evaluation of drug taste masking effect by electronic tongue. Mod. Traditional Chin. Med. Materia Medica-World Sci. Technol. 15 (7), 1532–1537. 10.11842/wst.2013.07.008

[B61] Li X. Wu Z. Liu R. Xu Z. Shi J. Li H. (2011). Study on bitterness evaluation of Chinese.

[B39] Li X. Wu Z. Liu R. Xu Z. Shi J. Li H. (2011). Study on bitterness evaluation of Chinese Herbal Decoction by THTPM. Chin. J. Exp. Traditional Med. Formulae 17 (23), 11–13. 10.13422/j.cnki.syfjx.2011.23.017.%W.CNKI

[B62] Lin Z. Zhang Q. Liu R. Gao X. Zhang L. Kang B. (2016). Evaluation of the bitterness of traditional Chinese medicines using an E-tongue coupled with a robust partial least squares regression method. Sensors (Basel) 16 (2), 151. 10.3390/s16020151 26821026 PMC4801529

[B63] Liu D. T. Besser G. Oeller F. Mueller C. A. Renner B. (2020). Bitter taste perception of the human tongue mediated by quinine and caffeine impregnated taste strips. Ann. Otol. Rhinol. Laryngol. 129 (8), 813–820. 10.1177/0003489420906187 32028784 PMC7357182

[B64] Liu R. Zhang X. Li X. Shi J. Li H. Qiu J. (2013). Drug evaluation of bitterness intensity by three kinds of THTPM. Chin. J. Exp. Tradit. Med. Formulae 19 (20), 118–122. 10.7501/j.issn.0253-2670.2013.16.009

[B65] Liu R. Li X. Yao j. Gui x. Wang Q. Shi J. (2012). A drug bitterness measurement method based on bitterness threshold concentration.

[B66] Liu R. Wang Y. Tian L. Gui X. Shi J. Zhang L. (2019). Masking efficiency and regularity of bitterness suppressants to berberine hydrochloride based on tongue taste and electronic tongue taste. Chin. Pharm. J. 54 (03), 208–218. 10.11669/cpj.2019.03.008

[B67] Liu R. Zhang X. Li X. Shi J. Li H. Qiu J. (2013). Drug evaluation of bitterness intensity by three kinds of THTPM. Chin. J. Exp. Traditional Med. Formulae 19 (20), 118–122.

[B68] Liu R. Zhang X. Zhang L. Gao X. Li H. Shi J. (2014a). Bitterness intensity prediction of berberine hydrochloride using an electronic tongue and a GA-BP neural network. Exp. Ther. Med. 7 (6), 1696–1702. 10.3892/etm.2014.1614 24926369 PMC4043613

[B69] Li Xuelin Z. X. Liu R. Huiling Li Jixi Q. Wu Z. (2013). Study on quantitation of bitterness intensity and relationship between bitterness intensity and concentration of bitter drug. World Sci. Technology/Modernization Traditional Chin. Med. Materia Medica (04), 667–671.

[B70] Loney G. C. Blonde G. D. Eckel L. A. Spector A. C. (2012). Determinants of taste preference and acceptability: quality versus hedonics. J. Neurosci. 32 (29), 10086–10092. 10.1523/jneurosci.6036-11.2012 22815522 PMC3422882

[B71] Loney G. C. Torregrossa A. M. Smith J. C. Sclafani A. Eckel L. A. (2011). Rats display a robust bimodal preference profile for sucralose. Chem. Senses 36 (8), 733–745. 10.1093/chemse/bjr048 21653913 PMC3175105

[B72] Ma Z. Taruno A. Ohmoto M. Jyotaki M. Lim J. C. Miyazaki H. (2018). CALHM3 is essential for rapid ion channel-mediated purinergic neurotransmission of GPCR-mediated tastes. Neuron 98 (3), 547–561.e10. 10.1016/j.neuron.2018.03.043 29681531 PMC5934295

[B73] Maniruzzaman M. Bonnefille M. Aranyos A. Snowden M. J. Douroumis D. (2014). An *in-vivo* and *in-vitro* taste masking evaluation of bitter melt-extruded drugs. J. Pharm. Pharmacol. 66 (2), 323–337. 10.1111/jphp.12169 24180412

[B74] Maniruzzaman M. Douroumis D. (2015). An in-vitro-in-vivo taste assessment of bitter drug: comparative electronic tongues study. J. Pharm. Pharmacol. 67 (1), 43–55. 10.1111/jphp.12319 25182780

[B75] Margulis E. Dagan-Wiener A. Ives R. S. Jaffari S. Siems K. Niv M. Y. (2021). Intense bitterness of molecules: machine learning for expediting drug discovery. Comput. Struct. Biotechnol. J. 19, 568–576. 10.1016/j.csbj.2020.12.030 33510862 PMC7807207

[B76] Medicine C. A. o.C. (2024). Technical specification for sensory evaluation of bitterness of oral liquid preparation of traditional Chinese medicine. T/CACM 1574—2024.

[B77] Mennella J. A. Pepino M. Y. Duke F. F. Reed D. R. (2011). Psychophysical dissection of genotype effects on human bitter perception. Chem. Senses 36 (2), 161–167. 10.1093/chemse/bjq106 20980355 PMC3020389

[B78] Meyerhof W. (2005). Elucidation of mammalian bitter taste. Rev. Physiol. Biochem. Pharmacol. 154, 37–72. 10.1007/s10254-005-0041-0 16032395

[B79] Meyerhof W. Batram C. Kuhn C. Brockhoff A. Chudoba E. Bufe B. (2010). The molecular receptive ranges of human TAS2R bitter taste receptors. Chem. Senses 35 (2), 157–170. 10.1093/chemse/bjp092 20022913

[B80] Milne C. P. Bruss J. B. (2008). The economics of pediatric formulation development for off-patent drugs. Clin. Ther. 30 (11), 2133–2145. 10.1016/j.clinthera.2008.11.019 19108801

[B81] Miyanaga Y. Inoue N. Ohnishi A. Fujisawa E. Yamaguchi M. Uchida T. (2003). Quantitative prediction of the bitterness suppression of elemental diets by various flavors using a taste sensor. Pharm. Res. 20 (12), 1932–1938. 10.1023/b:pham.0000008039.59875.4f 14725356

[B82] Münster M. Mohamed-Ahmed A. H. A. Immohr L. I. Schoch C. Schmidt C. Tuleu C. (2017). Comparative *in vitro* and *in vivo* taste assessment of liquid praziquantel formulations. Int. J. Pharm. 529 (1-2), 310–318. 10.1016/j.ijpharm.2017.06.084 28689966

[B83] Mura E. Yagi M. Yokota K. Seto E. Matsumiya K. Matsumura Y. (2018). Tolerance of bitter stimuli and attenuation/accumulation of their bitterness in humans. Biosci. Biotechnol. Biochem. 82 (9), 1539–1549. 10.1080/09168451.2018.1484273 29912652

[B84] Mustafa A. M. Caprioli G. Ricciutelli M. Maggi F. Marín R. Vittori S. (2015). Comparative HPLC/ESI-MS and HPLC/DAD study of different populations of cultivated, wild and commercial Gentiana lutea L. Food Chem. 174, 426–433. 10.1016/j.foodchem.2014.11.089 25529701

[B85] Nakamura H. Uchida S. Sugiura T. Namiki N. (2015). The prediction of the palatability of orally disintegrating tablets by an electronic gustatory system. Int. J. Pharm. 493 (1-2), 305–312. 10.1016/j.ijpharm.2015.07.056 26216412

[B86] Nakamura T. Akiyoshi T. Tanaka N. Shinozuka K. Matzno S. Nakabayashi T. (2003). Effect of quinine solutions on intracellular Ca2+ levels in neuro-2a cells--conventional physiological method for the evaluation of bitterness. Biol. Pharm. Bull. 26 (11), 1637–1640. 10.1248/bpb.26.1637 14600419

[B87] Narukawa M. Noga C. Ueno Y. Sato T. Misaka T. Watanabe T. (2011). Evaluation of the bitterness of green tea catechins by a cell-based assay with the human bitter taste receptor hTAS2R39. Biochem. Biophys. Res. Commun. 405 (4), 620–625. 10.1016/j.bbrc.2011.01.079 21272567

[B88] Noble A. C. (1994). Bitterness in wine. Physiol. Behav. 56 (6), 1251–1255. 10.1016/0031-9384(94)90373-5 7878098

[B89] Nolden A. A. Feeney E. L. (2020). Genetic differences in taste receptors: implications for the food industry. Annu. Rev. Food Sci. Technol. 11, 183–204. 10.1146/annurev-food-032519-051653 31922882

[B90] Noorjahan A. Amrita B. Kavita S. (2014). *In vivo* evaluation of taste masking for developed chewable and orodispersible tablets in humans and rats. Pharm. Dev. Technol. 19 (3), 290–295. 10.3109/10837450.2013.778870 23514223

[B91] Nordenmalm S. Kimland E. Ligas F. Lehmann B. Pelle B. Nafria B. (2019). Children's views on taking medicines and participating in clinical trials. Archives Dis. Child. 104 (9), 900–905. 10.1136/archdischild-2018-316511 31201156

[B92] Ogi K. Yamashita H. Terada T. Homma R. Shimizu-Ibuka A. Yoshimura E. (2015). Long-chain fatty acids elicit a bitterness-masking effect on quinine and other nitrogenous bitter substances by formation of insoluble binary complexes. J. Agric. Food Chem. 63, 8493–8500. 10.1021/acs.jafc.5b03193 26365517

[B93] Omür-Ozbek P. Dietrich A. M. (2008). Developing hexanal as an odor reference standard for sensory analysis of drinking water. Water Res. 42 (10-11), 2598–2604. 10.1016/j.watres.2008.01.010 18280533

[B94] Park H.-W. Kim M.-J. Seo S. Yoo S. Hong J.-H. (2017). Relative sweetness and sweetness quality of Xylobiose. Food Sci. Biotechnol. 26 (3), 689–696. 10.1007/s10068-017-0109-z 30263593 PMC6049597

[B95] Podrażka M. Bączyńska E. Kundys M. Jeleń P. S. Witkowska Nery E. (2017). Electronic tongue-A tool for all tastes? Biosens. (Basel) 8 (1), 3. 10.3390/bios8010003 PMC5872051 29301230

[B96] Polshin E. Rudnitskaya A. Kirsanov D. Legin A. Saison D. Delvaux F. (2010). Electronic tongue as a screening tool for rapid analysis of beer. Talanta 81 (1-2), 88–94. 10.1016/j.talanta.2009.11.041 20188892

[B97] Qingjun L. Ping W. (2009). Cell-based biosensors: principles and applications. Boston, London: Artech.

[B98] Ranmal S. R. Nhouchi Z. Keeley A. Adler L. Lavarde M. Pensé-Lhéritier A. M. (2023). Taste assessment for paediatric drug Development: a comparison of bitterness taste aversion in children versus Naïve and expert young adult assessors. Int. J. Pharm. 647, 123494. 10.1016/j.ijpharm.2023.123494 37806503

[B99] Roper S. D. (2007). Signal transduction and information processing in mammalian taste buds. Pflugers Arch. 454 (5), 759–776. 10.1007/s00424-007-0247-x 17468883 PMC3723147

[B100] Roudnitzky N. Behrens M. Engel A. Kohl S. Thalmann S. Hübner S. (2015). Receptor polymorphism and genomic structure interact to shape bitter taste perception. PLoS Genet. 11 (9), e1005530. 10.1371/journal.pgen.1005530 26406243 PMC4583475

[B101] Roudnitzky N. Bufe B. Thalmann S. Kuhn C. Gunn H. C. Xing C. (2011). Genomic, genetic and functional dissection of bitter taste responses to artificial sweeteners. Hum. Mol. Genet. 20 (17), 3437–3449. 10.1093/hmg/ddr252 21672920

[B102] Rudnitskaya A. Kirsanov D. Blinova Y. Legin E. Seleznev B. Clapham D. (2013). Assessment of bitter taste of pharmaceuticals with multisensor system employing 3 way PLS regression. Anal. Chim. Acta 770, 45–52. 10.1016/j.aca.2013.02.006 23498685

[B103] Rudnitskaya A. Nieuwoudt H. H. Muller N. Legin A. du Toit M. Bauer F. F. (2010). Instrumental measurement of bitter taste in red wine using an electronic tongue. Anal. Bioanal. Chem. 397 (7), 3051–3060. 10.1007/s00216-010-3885-3 20549490

[B104] Ruixin L. Huiling L. Xuelin L. Xingfen Z. Jixi Q. (2013). Evaluation on taste-masking effect of Andrographis Herba by electronic tongue. Chin. Traditional Herb. Drugs 44 (16), 2240–2245. 10.7501/j.issn.0253-2670.2013.16.009

[B105] Sakurai T. Misaka T. Nagai T. Ishimaru Y. Matsuo S. Asakura T. (2009). pH-Dependent inhibition of the human bitter taste receptor hTAS2R16 by a variety of acidic substances. J. Agric. Food Chem. 57 (6), 2508–2514. 10.1021/jf8040148 19231899

[B106] Schalk P. Kohl M. Herrmann H. J. Schwappacher R. Rimmele M. E. Buettner A. (2018). Influence of cancer and acute inflammatory disease on taste perception: a clinical pilot study. Support Care Cancer 26 (3), 843–851. 10.1007/s00520-017-3898-y 28948404 PMC5785616

[B107] Schienle A. Schlintl C. (2020). The association between quinine hydrochloride sensitivity and disgust proneness in children and adults. Springer U. S. 13 (1), 78–83. 10.1007/s12078-019-09268-6

[B108] Shah P. P. Mashru R. C. (2008). Formulation and evaluation of taste masked oral reconstitutable suspension of primaquine phosphate. AAPS PharmSciTech 9 (3), 1025–1030. 10.1208/s12249-008-9137-6 18770047 PMC2977032

[B109] Shi J. Zhang X. Qiu J. Li X. Liu R. (2013). Investigation of bitter masking mechanism of β-cyclodextrin to several traditional Chinese medicines. Chin. J. Exp. Traditional Med. Formulae 19 (12). 10.11653/syfj2013120001

[B110] Shibamoto T. Harada K. Mihara S. Nishimura O. Yamaguchi K. Aitoku A. (1981). APPLICATION OF HPLC FOR EVALUATION OF COFFEE FLAVOR QUALITY. Qual. Foods and Beverages, 311–334. 10.1016/b978-0-12-169102-8.50028-3

[B111] Sook Chung H. Lee S. Y. (2012). Modification of ginseng flavors by bitter compounds found in chocolate and coffee. J. Food Sci. 77 (6), S202–S210. 10.1111/j.1750-3841.2012.02716.x 22591221

[B112] Soto J. (2016). “Assessing the feasibility of using an animal model for *in vivo* taste assessment of pharmaceutical compounds and formulations,” in Ucl.

[B113] Suryawanshi S. Mehrotra N. Asthana R. K. Gupta R. C. (2006). Liquid chromatography/tandem mass spectrometric study and analysis of xanthone and secoiridoid glycoside composition of Swertia chirata, a potent antidiabetic. Rapid Commun. Mass Spectrom. 20 (24), 3761–3768. 10.1002/rcm.2795 17120271

[B114] Tomlinson J. B. Ormrod I. H. L. Sharpe F. R. (2013). A NOVEL METHOD FOR BITTERNESS DETERMINATION IN BEER USING A DELAYED FLUORESCENCE TECHNIQUE. J. Inst. Brew. 101 (2), 113–118. 10.1002/j.2050-0416.1995.tb00855.x

[B115] Torrico D. D. Sae-Eaw A. Sriwattana S. Boeneke C. Prinyawiwatkul W. (2015). Oil-in-Water emulsion exhibits bitterness-suppressing effects in a sensory threshold study. J. Food Sci. 80 (6), S1404–S1411. 10.1111/1750-3841.12901 25968872

[B116] Uchida T. Kobayashi Y. Miyanaga Y. Toukubo R. Ikezaki H. Taniguchi A. (2001). A new method for evaluating the bitterness of medicines by semi-continuous measurement of adsorption using a taste sensor. Chem. Pharm. Bull. (Tokyo) 49 (10), 1336–1339. 10.1248/cpb.49.1336 11605665

[B117] Wang H. (2022a). *Research on basic taste perception recognition based on physiological electrical signals.* Doctor.

[B118] Wang Q. Gao X. Gui X. Wang Y. Wang J. Li C. (2022). Study on quantitative method of equivalent molecular bitterness of traditional Chinese medicines based on bitterness threshold concentration. Chin. Traditional Herb. Drugs 53 (21), 6698–6705. 10.7501/j.issn.0253-2670.2022.21.006

[B119] Wang Y. Chen P. Gui X. Yao J. Zhang L. Shi J. (2021). Study on four kinds of taste classification and identification of natural medicines based on electronic tongue. China J. Traditional Chin. Med. Pharm. 36 (01), 423–433.

[B120] Wang Y. Feng Y. Wu Y. Liang S. Xu D. (2013). Sensory evaluation of the taste of berberine hydrochloride using an Electronic Tongue. Fitoterapia 86, 137–143. 10.1016/j.fitote.2013.02.010 23481282

[B122] Yanagisawa T. Misaka T. (2021). Characterization of the human bitter taste receptor response to sesquiterpene lactones from edible asteraceae species and suppression of bitterness through pH control. ACS Omega 6 (6), 4401–4407. 10.1021/acsomega.0c05599 33644553 PMC7906577

[B123] Yang J. Qiu M. Lu T. Yang S. Yu J. Lin J. (2023). Discovery and verification of bitter components in Panax notoginseng based on the integrated strategy of pharmacophore model, system separation and bitter tracing technology. Food Chem. 428, 136716. 10.1016/j.foodchem.2023.136716 37413835

[B124] Yang P. Liu Q. Metaxas D. N. (2010). “RankBoost with l1 regularization for facial expression recognition and intensity estimation,” in IEEE International Conference on Computer Vision.

[B125] Yang Z. xin Meng Y. Wang Q. Yang B. Kuang H. (2011). Substance basis of bitter resolution and composition from fructus evodiae. Chin. J. Exp. Traditional Med. Formulae 17 (021), 74–77. 10.13422/j.cnki.syfjx.2011.21.029

[B126] Yewale C. P. Rathi M. N. Kore G. G. Jadhav G. V. Wagh M. P. (2013). Formulation and development of taste masked fast-disintegrating tablets (FDTs) of Chlorpheniramine maleate using ion-exchange resins. Pharm. Dev. Technol. 18 (2), 367–376. 10.3109/10837450.2011.627870 22023351

[B127] Yoneda T. Saitou K. Mizushige T. Matsumura S. Manabe Y. Tsuzuki S. (2007). The palatability of corn oil and linoleic acid to mice as measured by short-term two-bottle choice and licking tests. Physiol. Behav. 91 (2-3), 304–309. 10.1016/j.physbeh.2007.03.006 17459430

[B128] Yu M. Li T. Raza A. Wang L. Song H. Zhang Y. (2020). Sensory-guided identification of bitter compounds in hangbaizhi (angelica dahurica). Food Res. Int. 129, 108880. 10.1016/j.foodres.2019.108880 32036877

[B129] Zeng G. (1990). The taste change trend when the molecular structure of the flavor is changed. Chin. J. Nat. 13 (2), 70–75.

[B130] Zeng Y. Guo L. Wang J. Huang L. Tian Z. Jiao L. (2015). Study on taste information of different Scutellaria baicalensis georgi and correlation between taste information and main chemical compositions based on technology of electronic-tongue. Mod. Chin. Med. 17(11), 1139–1147. 10.13313/j.issn.1673-4890.2015.11.007

[B131] Zhang Pu Zhang Y. Gui X. Shi J. Zhang H. Feng W. (2021). Study on superposition rule of bitterness of decoction of Chinese materia medica based on traditional human taste panel method and electronic tongue method. Chin. Traditional Herb. Drugs 52 (3), 653–668. 10.7501/j.issn.0253-2670.2021.03.007

[B132] Zheng X. Wu F. Hong Y. Shen L. Lin X. Feng Y. (2018). Developments in taste-masking techniques for traditional Chinese medicines. Pharmaceutics 10 (3), 157. 10.3390/pharmaceutics10030157 30213035 PMC6161181

[B133] Zhi R. Cao L. Cao G. (2017). Asians' facial responsiveness to basic tastes by automated facial expression analysis system. J. Food Sci. 82 (3), 794–806. 10.1111/1750-3841.13611 28140464

[B134] Zuluaga G. (2024). Potential of bitter medicinal plants: a review of flavor physiology. Pharm. (Basel) 17 (6), 722. 10.3390/ph17060722 PMC11206615 38931389

