# Supplementary material for: One of the major challenges of masking the bitter taste in medications: an overview of quantitative methods for bitterness
Source: Front Chem. 2024 Aug 14;12:1449536. doi: 10.3389/fchem.2024.1449536 (PMC11349634; doi:10.3389/fchem.2024.1449536)
Supplement: Supplementary file 1 [file DataSheet1.PDF]

Supplementary table 1 Taste of natural drugs with only one bitter taste

No.	Name of natural medicine	Taste situation	No.	Name of natural medicine	Taste situation	No.	Name of natural medicine	Taste situation
1	GENTIANAE RADIX ET RHIZOMA	very bitter	46	CORYDALIS DECUMBENTIS RHIZOMA	bitter	91	OLIBANUM	slightly bitter
2	SOPHORAE TONKINENSIS RADIX ET RHIZOMA	acridity	47	LAGGERAE HERBA	bitter	92	ARTEMISIAE SCOPARIAE HERBA	slightly bitter
3	STRYCHNI SEMEN	acridity	48	AKEBIAE FRUCTUS	bitter	93	TRIGONELLAE SEMEN	slightly bitter
4	STRYCHNI SEMEN PULVERATUM	acridity	49	SCUTELLARIAE RADIX	bitter	94	ILICIS CORNUTAE FOLIUM	slightly bitter
5	PHELLODENDRI AMURENSIS CORTEX	acridity	50	FIBRAUREAE CAULIS	bitter	95	CYPERI RHIZOMA	slightly bitter
6	GENTIANAE RHODANTHAE HERBA	acridity	51	DICHROAE RADIX	bitter	96	LAGOTIDIS HERBA	slightly bitter
7	ALOE	acridity	52	CHRYSANTHEMI INDICI FLOS	bitter	97	TRACHELOSPERMI CAULIS ET FOLIUM	slightly bitter
8	PICRASMAE RAMULUS ET FOLIUM	acridity	53	SUIS FELLIS PULVIS	bitter	98	NELUMBINIS FOLIUM	slightly bitter
9	SOPHORAE FLAVESCENTIS RADIX	acridity	54	AJUGAE HERBA	bitter	99	PERSICAE SEMEN	slightly bitter
10	CONYZAE HERBA	acridity	55	FRITILLARIAE HUPEHENSIS BULBUS	bitter	100	BUPLEURI RADIX	slightly bitter

11	PICRORHIZAE RHIZOMA	acridity	56	AILANTHI CORTEX	bitter	101	PEGAEOPHYTI RADIX ETRHIZOMA	slightly bitter
12	BRUCEAE FRUCTUS	acridity	57	SOPHORAE FRUCTUS	bitter	102	LEONURI HERBA	slightly bitter
13	ANDROGRAPHIS HERBA	acridity	58	SYRINGAE CORTEX	bitter	103	FRITILLARIAE THUNBERGII BULBUS	slightly bitter
14	COPTIDIS RHIZOMA	acridity	59	CITRI RETICULATAE SEMEN	bitter	104	DIOSCOREA PANTHAICAE RHIZOMA	slightly bitter
15	PHELLODENDRI CHINENSIS CORTEX	acridity	60	PTEROCEPHALI HERBA	bitter	105	CICHORII HERBA CICHORII RADIX	slightly bitter
16	BERBERIDIS RADIX	bitter	61	IMPERATAE RHIZOMA	bitter	106	GINKGO FOLIUM	slightly bitter
17	TURPINIAE FOLIUM	bitter	62	CELOSIAE CRISTATAE FLOS	bitter	107	INULAE FLOS	slightly bitter
18	SENECIONIS SCANDENTIS HEBRA	bitter	63	EUCOMMIAE CORTEX	A little bitter	108	DIOSCOREAE SPONGIOSAE RHIZOMA	slightly bitter
19	VLADIMIRIAE RADIX	bitter	64	BOLBOSTEMMATIS RHIZOMA	slightly bitter	109	POLYGONI AVICULARIS HERBA	slightly bitter
20	VERBENAE HERBA	bitter	65	FRITILLARIAE CIRRHOSAE BULBUS	slightly bitter	110	VIOLAE HERBA	slightly bitter
21	EUPOLYPHAGA STELEOPHAGA	bitter	66	POGOSTEMONIS HERBA	slightly bitter	111	SENNAE FOLIUM	slightly bitter
22	MAHONIAE CAULIS	bitter	67	CIRSII HERBA	slightly bitter	112	ACHILLEAE HERBA	slightly bitter

23	ARTEMISIAE ARGYI FOLIUM	bitter	68	SAUSSUREAE INVOLUCRATAE HERBA	slightly bitter	113	TARAXACI HERBA	slightly bitter
24	LYSIONOTI HERBA	bitter	69	TRICHOSANTHIS RADIX	slightly bitter	114	SOPHORAE FLOS	slightly bitter
25	FRITILLARIAE USSURIENSIS BULBUS	bitter	70	AUCKLANDIAE RADIX	slightly bitter	115	OMPHALIA	slightly bitter
26	MENISPERMI RHIZOMA	bitter	71	OROXYLI SEMEN	slightly bitter	116	SIEGESBECKIAE HERBA	slightly bitter
27	BLETILLAE RHIZOMA	bitter	72	AGRIMONIAE HERBA	slightly bitter	117	RHAPONTICI RADIX	slightly bitter
28	SWERTIAE HERBA	bitter	73	CHELIDONII HERBA	slightly bitter	118	VISCI HERBA	slightly bitter
29	CORYDALIS RHIZOMA	bitter	74	DICTAMNI CORTEX	slightly bitter	119	CARPESII FRUCTUS	slightly bitter
30	CRINIS CARBONISATUS	bitter	75	CYNANCHI ATRATI RADIX ET RHIZOMA	slightly bitter	120	PLANTAGINIS HERBA	slightly bitter
31	STEPHANIAE TETRANDRAE RADIX	bitter	76	SCUTELLARIAE BARBATAE HERBA	slightly bitter	121	CORDYCEPS	slightly bitter
32	FORSYTHIAE FRUCTUS	bitter	77	KOCHIAE FRUCTUS	slightly bitter	122	CROCI STIGMA	slightly bitter
33	AQUILARIAE LIGNUM RESINATUM	bitter	78	FRITILLARIAE PALLIDIFLORAE BULBUS	slightly bitter	123	LILII BULBUS	slightly bitter
34	SINOMENII CAULIS	bitter	79	CASSIAE SEMEN	slightly bitter	124	HORDEI FRUCTUS GERMINATUS	slightly bitter

35	SWERTIAE MILEENSIS HERBA	bitter	80	ERIGERONTIS HERBA	slightly bitter	125	EUCOMMIAE FOLIUM	slightly bitter
36	PICRIAE HERBA	bitter	81	CARTHAMI FLOS	slightly bitter	126	EPIMEDII WUSHANENSIS FOLIUM	slightly bitter
37	CORYDALIS BUNGEANAE HERBA	bitter	82	XANTHII FRUCTUS	slightly bitter	127	LONICE RAEJAPONICAE CAULIS	slightly bitter
38	ARMENIACAE SEMEN AMARUM	bitter	83	GLECHOMAE HERBA	slightly bitter	128	SEDI HERBA	slightly bitter
39	MELIAE CORTEX	bitter	84	SETARIAE FRUCTUS GERMINATUS	slightly bitter	129	EUPATORII HERBA	slightly bitter
40	TINOSPORAE RADIX	bitter	85	ABRI HERBA	slightly bitter	130	SELAGINELLAE HERBA	slightly bitter
41	LEONURI FRUCTUS	bitter	86	ARTEMISIAE ANNUAE HERBA	slightly bitter	131	ALISMATIS RHIZOMA	slightly bitter
42	PERIPLOCAE CORTEX	bitter	87	ERIOBOTRYAE FOLIUM	slightly bitter	132	DALBERGIAE ODORIFERAE LIGNUM	slightly bitter
43	ARISAEMA CUM BILE	bitter	88	PRUNI SEMEN	slightly bitter	133	DATURAEFLOS	slightly bitter
44	FRAXINI CORTEX	bitter	89	CURCUMAE RADIX	slightly bitter			
45	NELUMBINIS PLUMULA	bitter	90	INULAE HERBA	slightly bitter			

Supplementary table 2 The taste of natural medicines with bitter taste and other tastes

No.	Name of natural medicine	Taste situation	No.	Name of natural medicine	Taste situation	No.	Name of natural medicine	Taste situation
1	SOLIDAGINIS HERBA	slightly bitter、pungent	61	POLYGONI MULTIFLORI RADIX	slightly bitter、sweet、astringent	121	FARFARAE FLOS	slightly bitter and pungent
2	BOVIS CALCULUS ARTIFACTUS	bitter，slightly sweet	62	POLYGONI MULTIFLORI RADIX PRAEPARATA	slightly sweet、bitter、astringent	122	DESCURAINIAE SEMEN LEPIDII SEMEN	slightly pungent、bitter
3	GINSENG RADIX ET RHIZOMA	slightly bitter、pungent	63	CITRI SARCODACTYLIS FRUCTUS	Sweet after bitter	123	TRACHYCARPI PETIOLUS	bitter、astringent
4	GINSENG FOLIUM	bitter、sweet	64	MAGNOLIAE FLOS	pungent-cool and A little bitter	124	PEUCEDANI DECURSIVI RADIX	slightly bitter、pungent
5	CATECHU	astringent、bitter	65	NOTOPTERYGII RHIZOMA ET RADIX	slightly bitter and pungent	125	CALLICARPAE FORMOSANAE FOLIUM	slightly bitter、astringent
6	MURRAYAE FOLIUM ET CACUMEN	pungent、slightly bitter	66	MYRRHA	bitter and slightly pungent	126	ASTERIS RADIX ET RHIZOMA	sweet、slightly bitter
7	NOTOGINSENG RADIX ET	Taste bitter	67	PSORALEAE FRUCTUS	pungent、slightly	127	GEI HERBA	pungent、

	RHIZOMA	back sweet			bitter			slightly bitter
8	SPARGANII RHIZOMA	pungent 、 bitter, mild	68	CITRI RETICULATAE PERICARPIUM	pungent、 bitter	128	TRIBULI FRUCTUS	bitter、pungent
9	ZINGIBERIS RHIZOMA	slightly bitter、 slightly spicy	69	CITRI RETICULATAE PERICARPIUM VIRIDE	bitter、pungent	129	PROPOLIS	slightly bitter、 slightly astringent, slightly numb and spicy.
10	INULAE RADIX	bitter、pungent	70	ROSAE RUGOSAE FLOS	slightly bitter and astringent	130	PHYSALIS CALYX SEU FRUCTUS	The calyx tastes bitter, the fruit tastes sweet and slightly sour.
11	PSEUDOLARICIS CORTEX	slightly bitter and astringent	71	ISATIDIS RADIX	Bitter and astringent after slightly sweet	131	RHODODENDRI DAURICI FOLIUM	more bitter 、 slightly pungent
12	CALLICARPAE MACROPHYLLAE FOLIUM	pungent 、 slightly bitter	72	ACANTHOPANACIS SENTICOSI RADIX ET RHIZOMA SEU CAULIS	slightly pungent、 A little bitter、 astringent	132	KADSURAE CAULIS	bitter and astringent
13	ISATIDIS FOLIUM	slightly sour、 bitter 、 astringent	73	POLYGONI CUSPIDATI RHIZOMA ET RADIX	slightly bitter、 astringent	133	CALLICARPAE NUDIFLORAE FOLIUM	astringent 、 slightly bitter

14	HALITUM	salt、slightly bitter and astringent	74	BERGENIAE RHIZOMA	bitter、astringent	134	POLYGONI TINCTORII FOLIUM	slightly astringent and a little bitter
15	RHEI RADIX ET RHIZOMA	bitter and slightly astringent	75	ANEMARRHENAE RHIZOMA	Slightly sweet, slightly bitter, chewed with viscosity	135	ENTADAE SEMEN	bitter
16	CIRSII JAPONICI HERBA CARBONISATA	bitter、astringent，cold	76	POTENTILLAE CHINENSIS HERBA	astringent、slightly bitter	136	ARECAE SEMEN	astringent、slightly bitter
17	LIRIOPES RADIX	sweet、slightly bitter	77	PLATYCLADI CACUMEN	bitter、astringent、slightly pungent	137	ARECAE SEMEN TOSTUM	astringent、slightly bitter
18	CALLICARPAE CAULIS ET FOLIUM	slightly bitter、astringent	78	LONICERAE JAPONICAE FLOS	light、slightly bitter	138	VALERIANAE JATAMANSI RHIZOMA ET RADIX	slightly bitter、pungent
19	HOMALOMENAE RHIZOMA	pungent、slightly bitter	79	EUPHORBIAE PEKINENSIS RADIX	slightly bitter and astringent	139	BELLADONNAE HERBA	slightly bitter、pungent
20	IRIDIS TECTORI RHIZOMA	sweet、bitter	80	PINI LIGNUM NODI	slightly bitter、pungent	140	CITRI EXOCARPIUM RUBRUM	slightly bitter、stingy
21	TOOSENDAN FRUCTUS	sour、bitter	81	RUBIAE RADIX ET RHIZOMA	slightly bitter, long chewing	141	CORNI FRUCTUS	sour、astringent、

					thorn tongue			slightly bitter
22	LIGUSTRI LUCIDI FRUCTUS	sweet 、slightlyly bitter and astringent	82	LITCHI SEMEN	slightly sweet、bitter、astringent	142	LONICERAE FLOS	slightly bitter、sweet
23	VACCARIAE SEMEN	slightly astringent 、bitter	83	CAROTAE FRUCTUS	slightly pungent、bitter	143	CRATAEGI FOLIUM	astringent 、slightly bitter
24	ASPARAGI RADIX	sweet、slightly bitter	84	AURANTII FRUCTUS	bitter 、 slightly sour	144	CHUANXIONG RHIZOMA	bitter、pungent, Slightly numb tongue, slightly back to the sweet
25	SEMIAQUILEGIAE RADIX	sweet、slightly bitter、pungent	85	AURANTII FRUCTUS IMMATURUS	bitter 、 slightly sour	145	SINOPODOPHYLLI FRUCTUS	sweet and sour, astringent ; the seed tastes bitter
26	AKEBIAE CAULIS	slightly bitter and astringent	86	GARDENIAE FRUCTUS	slightly sour and bitter	146	SCHISANDRAE CHINENSIS FRUCTUS	pungent 、slightly bitter
27	ACANTHOPANACIS CORTEX	slightlyly spicy and bitter	87	GARDENIAE FRUCTUS PRAEPARATUS	slightly sour and bitter	147	BOVIS CALCULUS	Bitter then sweet, have a cool feeling

28	ARCTII FRUCTUS	After bitter slightly spicy and slightly numb tongue	88	MAGNOLIAE OFFICINALIS CORTEX	spicy, slightly bitter	148	TERMINALIAE BELLIRICAE FRUCTUS	astringent 、 bitter
29	ACHYRANTHIS BIDENTATAE RADIX	slightly sweet and a little bitter	89	PHARBITIDIS SEMEN	pungent、 bitter	149	CIMICIFUGAE RHIZOMA	slightly bitter and astringent
30	WENYUJIN RHIZOMA CONCISUM	slightly bitter and pungent-cold	90	CITRI FRUCTUS	slightly weet and bitter 、pungent	150	ROSAE CHINENSIS FLOS	light 、 slightly bitter
31	CITRI GRANDIS EXOCARPIUM	bitter、slightly pungent	91	PARIDIS RHIZOMA	slightly bitter 、 stingy	151	LINDERAE RADIX	slightly bitter、pungent ， a sense of coolness
32	SALVIAE MILTIORRHIZAE RADIX ET RHIZOMA	slightly bitter and astringent	92	LAMIOPHLOMIS HERBA	slightly astringent、bitter	152	MUME FRUCTUS	slightly acid and bitter
33	PYRROSIAE FOLIUM	slightly bitter and astringent	93	ANGELICAE PUBESCENTIS RADIX	bitter、pungent、Micro hemp tongue	153	NARDOSTACHYOS RADIX ET RHIZOMA	bitter and pungent ， a sense of coolness
34	ACORI TATARINOWII RHIZOMA	bitter、slightly pungent	94	IMPATIENTIS SEMEN	light, slightly bitter	154	DENDROBII CAULIS	slightly bitter and sweet,

								chewed with viscosity
35	ILICIS CHINENSIS FOLIUM	bitter、astringent	95	CURCUMAE LONGAE RHIZOMA	bitter、pungent	155	GRANATI PERICARPIUM	bitter、astringent
36	PULSATILLAE RADIX	slightly bitter and astringent	96	PEUCEDANI RADIX	slightly bitter、pungent	156	CURCULIGINIS RHIZOMA	slightly bitter、pungent
37	PAEONIAE RADIX ALBA	slightly bitter、sour	97	DIOSCOREAE NIPPONICAE RHIZOMA	bitter、astringent	157	ANGELICAE DAHURICAE RADIX	pungent、slightly bitter
38	GINKGO SEMEN	sweet、slightly bitter	98	GENTIANAE MACROPHYLLAE RADIX	bitter、slightly astringent	158	LYCII CORTEX	sweet and then bitter
39	POLYGALAE JAPONICAE HERBA	slightly pungent、bitter	99	PANACIS MAJORIS RHIZOMA	slightly bitter、slightly sweet，chewing does not stab the throat	159	ANGELICAE SINENSIS RADIX	sweet、pungent、slightly bitter
40	RABDOSIAE RUBESCENTIS HERBA	bitter、sweet	100	CURCUMAE RHIZOMA	slightly bitter and pungent	160	CISTANCHES HERBA	sweet、slightly bitter
41	SCROPHULARIAE RADIX	sweet、slightly bitter	101	PLATYCODONIS RADIX	slightly sweet after bitter	161	ANEMONES RADDEANAE RHIZOMA	First light then slightly bitter and spicy
42	REHMANNIAE RADIX	slightly sweet、slightly bitter	102	PERSICAE RAMULUS	slightly bitter、astringent	162	BOVIS CALCULUS SATIVUS	bitter then sweet

43	SANGUISORBAE RADIX	slightly bitter 、astringent	103	BELAMCANDAE RHIZOMA	bitter 、 slightly pungent	163	GANODERMA	bitter and astringent
44	CISSAMPELOTIS HERBA	bitter、slightly sweet	104	BISTORTAE RHIZOMA	bitter、astringent	164	HYPERICI PERFORATI HERBA	slightly bitter and astringent
45	CHEBULAE FRUCTUS IMMATURUS	bitter	105	DIOSCOREAE HYPOGLAUCAE RHIZOMA	pungent、slightly bitter	165	SCHIZONEPETAE HERBA CARBONISATA	bitter and pungent
46	PANACIS QUINQUEFOLII RADIX	bitter 、astringent 、slightly sweet	106	ALPINIAE OXYPHYLLAE FRUCTUS	pungent、slightly bitter	166	SCHIZONEPETAE SPICA CARBONISATA	bitter and pungent
47	STEMONAE RADIX	sweet、bitter	107	AESCULI SEMEN	sweet after bitter	167	LITSEAE FRUCTUS	slightly spicy and slightly bitter
48	ARDISIAE CRENATAE RADIX	slightly bitter, thorn tongue feeling	108	PIPERIS KADSURAE CAULIS	slightly bitter 、 pungent	168	ALPINIAE KATSUMADAI SEMEN	pungent 、slightly bitter
49	PANACIS JAPONICI RHIZOMA	bitter, slightly sweet after	109	MARSDENIAE TENACISSIMAE CAULIS	bitter back sweet	169	TSAOKO FRUCTUS	pungent 、slightly bitter
50	PHYSOCHLAINAE RADIX	slightly bitter, slightly numb	110	MORI FOLIUM	light 、 slightly bitter and	170	AMOMI FRUCTUS	pungent-cold、slightly bitter

		tongue			astringent			
51	GINSENG RADIX ET RHIZOMA RUBRA	sweet、slightly bitter	111	SMILACIS CHINAE RHIZOMA	slightly bitter、astringent	171	POLYGONI MULTIFLORI CAULIS	slightly bitter 、astringent
52	RHODIOLAE CRENULATAE RADIX ET RHIZOMA	slightly bitter and astringent、sweet	112	CHRYSANTHEMI FLOS	sweet 、 slightly bitter	172	RAPHANI SEMEN	light 、 slightly bitter and pungent
53	OPHIOPOGONIS RADIX	sweet、slightly bitter	113	ILICIS ROTUNDAE CORTEX	bitter 、 slightly astringent	173	JUGLANDIS SEMEN	sweet；The seed coat tastes astringentt 、slightly bitter
54	POLYGALAE RADIX	bitter、slightly pungent	114	EUPATORII LINDLEYANI HERBA	bitter、astringent	174	CAMPSIS FLOS	slightly bitter、sour
55	PAEONIAE RADIX RUBRA	slightly bitter 、acor	115	STAUNTONIAE CAULIS ET FOLIUM	slightly bitter、astringent	175	MUME FLOS	slightly bitter、astringent
56	ATRACTYLODIS RHIZOMA	slightly sweet、pungent、bitter	116	EPHEDRAE HERBA	astringent 、slightly bitter	176	BUDDLEJAE FLOS	slightly bitter、pungent
57	ZANTHOXYLI RADIX	spicy and bitter tongue	117	PYROLAE HERBA	light 、 slightly bitter	177	ARNEBIAE RADIX	slightly bitter、astringent
58	EUODIAE FRUCTUS	spicy and bitter	118	CLINOPODII HERBA	astringent 、	178	CENTIPEDA HERBA	bitter、slightly

					slightly bitter			pungent
59	MOUTAN CORTEX	slightly bitter and astringent	119	DIPSACI RADIX	bitter、slightly sweet and then bitter	179	RICINI SEMEN	slightly bitter、pungent
60	VITICIS NEGUNDO FOLIUM	pungent 、 slightly bitter	120	DRYOPTERIDIS CRASSIRHIZOMATIS RHIZOMA	initially light and slightly astringent, then gradually bitter and pungent	180	LIGUSTICI RHIZOMA ET RADIX	pungent 、 bitter、slightly hemp
